# Gene regulation is governed by a core network in hepatocellular carcinoma

**DOI:** 10.1186/1752-0509-6-32

**Published:** 2012-05-01

**Authors:** Zuguang Gu, Chenyu Zhang, Jin Wang

**Affiliations:** 1 The State Key Laboratory of Pharmaceutical Biotechnology and Jiangsu Engineering Research Center for MicroRNA Biology and Biotechnology, School of Life Science, Nanjing University, Nanjing, 210093, China

## Abstract

**Background:**

Hepatocellular carcinoma (HCC) is one of the most lethal cancers worldwide, and the mechanisms that lead to the disease are still relatively unclear. However, with the development of high-throughput technologies it is possible to gain a systematic view of biological systems to enhance the understanding of the roles of genes associated with HCC. Thus, analysis of the mechanism of molecule interactions in the context of gene regulatory networks can reveal specific sub-networks that lead to the development of HCC.

**Results:**

In this study, we aimed to identify the most important gene regulations that are dysfunctional in HCC generation. Our method for constructing gene regulatory network is based on predicted target interactions, experimentally-supported interactions, and co-expression model. Regulators in the network included both transcription factors and microRNAs to provide a complete view of gene regulation. Analysis of gene regulatory network revealed that gene regulation in HCC is highly modular, in which different sets of regulators take charge of specific biological processes. We found that microRNAs mainly control biological functions related to mitochondria and oxidative reduction, while transcription factors control immune responses, extracellular activity and the cell cycle. On the higher level of gene regulation, there exists a core network that organizes regulations between different modules and maintains the robustness of the whole network. There is direct experimental evidence for most of the regulators in the core gene regulatory network relating to HCC. We infer it is the central controller of gene regulation. Finally, we explored the influence of the core gene regulatory network on biological pathways.

**Conclusions:**

Our analysis provides insights into the mechanism of transcriptional and post-transcriptional control in HCC. In particular, we highlight the importance of the core gene regulatory network; we propose that it is highly related to HCC and we believe further experimental validation is worthwhile.

## Background

Hepatocellular carcinoma (HCC) is the major histological subtype of liver cancer, and is among the most lethal cancers worldwide. The high cancer rates are especially found in the East, South-East Asia and sub-Saharan Africa [1]. Infection with hepatitis B (HBV) or C (HCV) viruses was found to be the main cause of the development of HCC in developing countries [1,2]. However, the current knowledge regarding the mechanisms of molecule interactions that lead to the disease pathogenesis is still quite limited [2].

With the development of high-throughput technologies such as microarray and next-generation sequencing, it is possible to create a systematic view of biological systems to improve our understanding of the roles of genes associated with diseases [3]. Since the abnormal state of proteins involved in diseases results from the altered expression of genes, analysis of the mechanisms of molecule interactions in the context of gene regulatory networks (GRNs) can reveal the specific sub-networks that lead to the dysfunction of regular biological systems [4].

GRNs are modelled as directed networks where interactions are directed from regulators to targets. Gene regulation is controlled by both transcription factors (TFs) and microRNAs (miRNAs). Transcription factors are proteins that bind to the promoter regions of target genes, and function by activating or inhibiting the expression of targets. For example, P53 [5], c-Myc [6] and E2F-1 [7] are frequently reported to be dysfunctional TFs in HCC. Moreover, miRNAs, a type of short non-coding RNAs, are involved in the post-transcriptional regulation of genes, either by degrading target mRNAs or by inhibiting the translation procedure [8,9]. It is known that miRNAs play a critical role in human cancer generation by various mechanisms [10,11]. Two representative miRNAs, miR-122 and miR-21, are highly expressed in liver tissue, where miR-122 is down-regulated and miR-21 is up-regulated in HCC [12]. One of the experimentally validated targets of miR-122 is Cyclin G1, thus, repression of miR-122 expression would enhance the cell cycle process and promote cell proliferation [13]. In turn, oncogenic miR-21 blocks the expression of apoptosis-related genes [14]. MiRNAs are transcribed from the genome contained in the nucleus, and hence expression of miRNAs is also regulated by TFs. As a result of mutual regulation by both miRNAs and TFs, gene regulation is assembled within the structure of a network.

Several studies have focused on the construction of GRNs. The first category of methods is the utilization of interactions from target predictions [15]. In this category, the relationships between TFs/miRNAs and their targets are predicted through sequence alignment or thermodynamics models. However, a major drawback of target prediction methods is the high false-positive rate, and as a result, GRNs constructed in this way contain a lot of noise. Therefore, analysis of GRNs can only provide the global attributes of the system, while the predictions for local regulations may not be reliable. The second category of methods is the integration of both target predictions and gene expression data. It can be regarded as the intersection of GRNs constructed by target predictions and GRNs constructed by co-expression models. Target prediction results only provide information regarding potential physical interactions between regulators and targets. The accuracy of the regulations can be validated by the correlations between regulators and targets on the expression level. However, utilizing expression data alone cannot fully capture the real regulations because correlations cannot elucidate whether the interactions are directly with the regulators or indirect. The most used methods are Pearson correlation [16] or multivariate linear regression model [17,18]. Additionally, with the rapid increase in the last few years in the validation of regulatory relationships using experimental approaches such as microarray, deep sequencing and ChIP-seq [19-21], these high quality data will no doubt contribute to the construction of networks.

In this study, we aimed to identify the most important gene regulations that are dysfunctional in HCC generation. Our model for GRN construction is based on predicted target interactions, experimentally-supported interactions and co-expression modeling. The network includes both TFs and miRNAs. We found the regulation strengths were quite different from TFs to miRNAs, thus cutoffs of correlation were set for TFs and miRNAs independently. A topological criterion was applied to select proper cutoffs for correlations in order to ensure that the final GRN made biological sense.

Analysis of the GRN revealed that gene regulation in HCC is highly modular, where different sets of regulators take charge of specific biological processes. We found that miRNAs mainly control biological functions related to mitochondria and oxidative reduction, while TFs control immune responses, extracellular activity and the cell cycle. On the higher level of gene regulation, there exists a core GRN that regulates different modules. The core GRN was critically important in maintaining the stability and robustness of the network. We postulate that it is the central controller of gene regulation in this context. In the core GRN, most of the regulators have been previously reported to relate to HCC, thus validating our findings. Finally, we explored the influence of the core GRN on biological pathways.

## Results and discussion

We focused on the dysfunction of gene regulation in HCC in which HBV is endemic. Microarray data was downloaded from the GEO database [GEO: GSE22058] [22], and genome-wide expression profiles of both miRNAs and mRNAs were examined.

### Network construction model

First, a candidate network was established by combining predicted target interactions and experimentally-supported interactions involving both TFs and miRNAs. Since this kind of network contains a lot of noise and does not relate to specific tissue, we re-filtered interactions using a co-expression model based on microarray data.

Co-expression models are frequently used to establish relationships between genes expressed in specific tissues [23]. In these models, if two genes share similar expression profiles, as measured by significant Pearson correlation coefficients, the two genes are connected in the network. In this step, we only calculated correlation coefficients between regulators and targets in the candidate network. If a TF/miRNA has similar or reversed expression patterns to some genes, then there is high probability that the TF/miRNA regulates these genes. Since the co-expression model cannot tell whether the regulation is direct or indirect, and interactions from the candidate network can only provide potential physical interactions, it is necessary for the integration of both data sources to provide stronger evidence for the gene regulations. Thus, the final GRN can be regarded as the intersection of the candidate network and the GRN constructed by the co-expression model. We first eliminated outliers in the expression profile data, and then calculated the correlation coefficients between regulators and targets using the Pearson method. The final regulations between regulators and targets must satisfy the following three conditions: 1) there exists a predicted target interaction or experimentally-supported interaction; 2) the correlation coefficient between miRNA and its targets should be negative; 3) the absolute value of the correlation coefficient is larger than the cutoff.

### Selection of cutoffs for correlation coefficients

**Figure 1:**
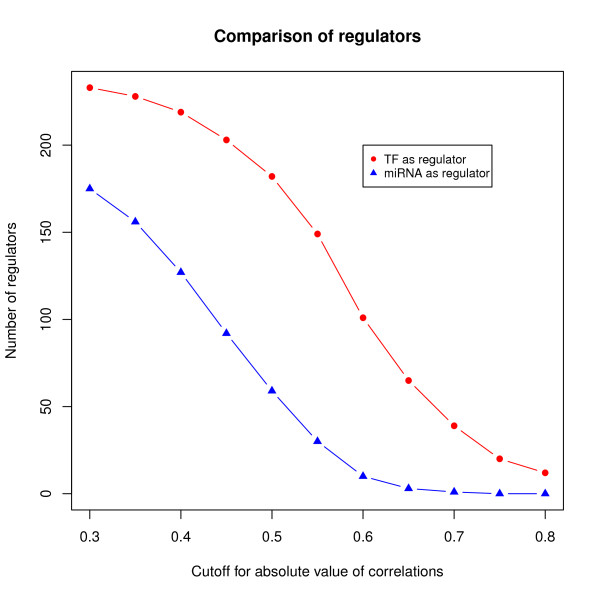
Number of regulators in the GRN, where only TFs or miRNAs are taken as regulators.

There are two types of regulators in the GRN, TFs and miRNAs, and we found that the regulation strength differs between the two. If the GRN is separated into a network where only TFs behave as regulators, and a network where only miRNAs are the regulator, under the same cutoff of correlation, the number of miRNAs is much less than that of TFs (Figure 1). For example, when the cutoff for the absolute value of correlation between the regulators and targets is set to 0.6, the number of TFs is 101, while only ten miRNAs are retained in the GRN. If we take the same cutoff for both TFs and miRNAs, there would be a high difference between the number of the two kinds of regulators, and the final GRN is highly biased in favor of TFs. The difference in the mechanism of TFs and miRNAs to regulate transcription is probably the reason for the different regulation strength. As a result, the cutoff for correlations of interactions where TFs are regulators and the cutoff for correlations of interactions where miRNAs are regulators were chosen independently.

The selection of cutoffs was processed through a topological criterion [24], which means the final GRN must be approximately scale-free. The scale-free network is common in biological networks where a very small amount of nodes connect to many neighbor nodes, while the remaining majority of nodes have extremely small connections [25]. The nodes with high numbers of connections are called hub nodes, and they are important within the network. It is known that some important TFs and miRNAs regulate many targets that result in cancer generation, and they are the hub regulators in GRNs.

**Figure 2:**
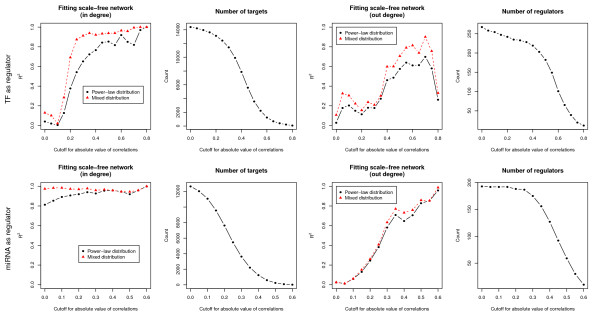
**Selection of cutoffs for correlations between regulators and targets by topological criterion.** The first row represents the GRN where only TFs are regulators, and the second row represents GRN where only miRNAs are regulators. The cutoff refers to the absolute value of correlation coefficient.

The characteristics of scale-free network are assessed from node degree distribution. The degree for a node is the number of neighbor nodes to which the node directly connects. In a scale-free network, the degree distribution is always represented as a power-law distribution [26] or exponential truncated power-law distribution [27]. We fitted the degree distribution of the GRN constructed from different cutoffs of correlation coefficients to power-law distribution and exponential truncated power-law distribution. The *R*^2^ value was used to measure the goodness-of-fit for these two distributions. Since the GRN is a directed network, the degree distribution is divided into in-degree distribution and out-degree distribution. Figure 2 illustrates how the cutoff for the absolute value of correlations affects *R*^2^ and the size of the GRN. For GRNs where only TFs are regulators, if no expression data are integrated (cutoff = 0), both of the in-degree distribution and out-degree distribution are completely not power-law (*R*^2^ ≈ 0). In other words, GRNs constructed only from candidate networks are not scale-free, and thus may not make biological sense. The same condition also occurs for the out-degree distribution of GRNs where only miRNAs are regulators. It highlights the importance for the use of expression data. In most circumstances, as the cutoff of the absolute correlation increases, *R*^2^ increases while the size of the GRN decreases, thus a trade-off between high *R*^2^ values and the correct size of the GRN is made. We chose cutoffs with the criterion that the *R*^2^ value first reaches a steady state for both in-degree distribution and out-degree distribution. In this study, the cutoff for the absolute value of correlation for interactions where TFs are regulators was set to 0.6, and that for miRNAs was set to 0.45.

### Network overview

**Figure 3:**
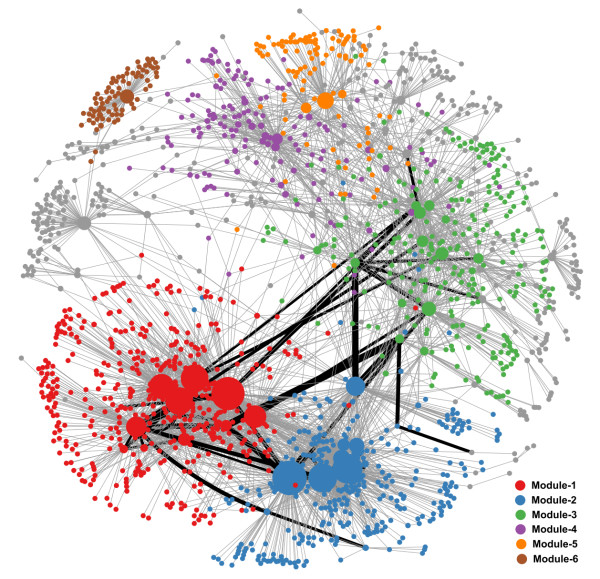
**The gene regulatory network in HCC.** Different colors represent nodes in different network modules. Size of nodes is proportional to the out-degree of nodes. Black edges represent regulations in the core GRN, and the width of the edges in the core GRN are proportional to the edge-betweenness values calculated from the global GRN.

After integrating predicted target interactions, experimentally-supported interactions and the co-expression model, the network was constructed with 1844 nodes. The biggest connected component contained 1691 nodes (91.7 % of all nodes) and was used for downstream analysis (Figure 3). The GRN constructed from the biggest connected component contained 80 miRNAs, 64 TFs, and 4199 interactions, which were composed of 1111 regulations from miRNAs to genes, 74 regulations from TFs to miRNAs and 3014 regulations from TFs to genes. Among the GRN, there were 484 interactions that were supported by experimental data. The complete adjacency list of the GRN can be found at Additional File 1.

### Network modules

**Figure 4:**
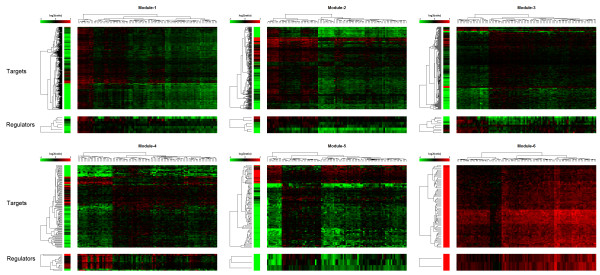
**Heatmap of expression values of genes in the six modules.** Heatmap of expression values of genes in the six modules identified in the network shown in Figure 3. For each figure, rows correspond to genes and columns correspond to samples in heatmaps. Expression values are logarithm of ratio value using base 2. The first column in front of each heat map is the *t*-value for each gene, and the color of the *t*-value represents whether the gene is up-regulated (red) or down-regulated (green). For each heatmap, the expression profile for targets and regulators are illustrated separately. Only expression for regulators listed in Table 1 is illustrated.

Genes in biological networks always have a structure in which genes are more closely connected [28]. This kind of sub-network is termed as a network module or community. We used walktrap algorithms [29] to find densely connected sub-networks where the absolute values of correlations were taken as the weight of edges. The six largest modules (covered 79.7 % of all nodes in GRN) are illustrated in Figure 3, and a summary of the six modules is listed in Table 1. Heatmaps of expression profile of regulators and targets in the six modules are illustrated in Figure 4.

**Table 1:** Summary of GRN modules

**Index**	**Size**	**Main regulators**	**Target gene functions**
1	419	RUNX3, RUNX2, POU2AF1, POU2F2, FLI1, BHLHB3, PRDM1	Immune response, Plasma membrane, Cell activation
2	328	HAND2, TCF4, FOXF1, FOXF2, ARID5B, FOXL1	Extracellular region, Cell adhension
3	270	miR-150, miR-142, miR-155, miR-181a, miR-342, miR-27a, miR-146a, miR-199a, miR-214, HNF4A	Mitochondrion, Oxidation reduction, Mitochondrial envelope
4	152	AR, miR-127, miR-377, miR-323, miR-299, miR-221, miR-433, miR-376a, miR-136, miR-18a, miR-296, miR-154, miR-431, miR-382, miR-369, miR-200b	Oxidation reduction, Cofactor metabolic process, Steroid metabolic process
5	103	NR1I3, NR1I2, ESR1	Oxidation reduction, Microsome, Fatty acid metabolic process
6	75	E2F1, E2F7	Cell cycle, Mitosis, Chromosome, Nuclear lumen
3, 4, 5	525	NR1I3,miR-150, miR-142, miR-155, miR-181a, AR, NR1I2, miR-342, miR-27a, miR-146a, HNF4A, miR-199a, miR-218, miR-214, miR-127, miR-132, ESR1, miR-377, SOX4, miR-323, miR-299, miR-221, miR-23a	Mitochondrion, Oxidation reduction, Cofactor binding,

Regulators are sorted by the number of targets. Regulators that regulate more than 80 % of genes in each module are listed. Gene Ontology enrichment was applied by DAVID to find common functions of genes. Size of each module corresponds to the number of nodes. The detailed enrichment results can be found in Additional File 2.

For HBV-induced HCC, the immune response of the host to clear abnormal pathogens is inhibited. Target genes in Module 1 are highly related to the immune response, and most of genes are down-regulated. There are seven main regulators in Module 1, in which RUNX3, POU2AF1, POU2F2, FLI1 and PRDM1 were significantly down-regulated (*p*-value < 1e-6). RUNX3 has been suggested to be a tumor suppressor, and its gene is frequently transcriptional silenced in cancer [30]. POU2F2, with its factor POU2AF1, acts as a cell survival factor in immune cells, and plays a central role in lymphoid-specific transcription of immunoglobulin genes [31]. FLI1 can affect apoptosis in tumor cells [32], and PRDM1 is a candidate tumor suppressor gene related to immune systems [33].

Cell adhesion is generally suppressed in cancers. Reduced cell adhesion allows cancer cells to disrupt the histological structure, resulting in the morphological features of malignant tumors [34]. Target genes in Module 2 related to extracellular activities are down-regulated. There are six main regulators in Module 2, in which HAND2, TCF4, FOXF1 and ARID5B (*p*-value < 1e-3) were significantly down-regulated, and FOXF2 was significantly up-regulated *(p*-value < 1e-4). HAND2 is reported to regulate extracellular matrix remodeling [35]. TCF4 is a key factor in the Wnt pathway and is involved in HCC cell proliferation [36]. FOXF1 deficiency was reported to decrease cell adhesion [37], and FOXF2 is important for extracellular matrix production [38].

The mitochondrion is a key organelle in cell metabolism. It is not only a power factory, but also regulates cell death pathways. In cancer cells, as a result of rapid proliferation, oxidative phosphorylation is suppressed in order that mitochondria consume less oxygen [39]. In our results, targets in Modules 3–5 are mostly related to the functions of mitochondria, such as oxidative reduction and metabolism. Among the regulators, miR-150, miR-146a, miR-199a, miR-214, together with NR1I3, AR, NR1I2 and ESR1 were significantly down-regulated *(p*-value < 1e-6) and miR-221 was significantly up-regulated (*p*-value < 1e-12). MiR-150 has been reported to inhibit liver cancer by negative regulation of c-Myb [40]. A polymorphism in miR-146a is associated with risk of HCC [41], while miR-199a induces apoptosis and inhibits the ERK pathway [42]. MiR-214 induces cell survival by targeting the PTEN/Akt pathway to suppress apoptosis [43], and mir-221 overexpression contributes to liver tumorigenesis [44]. Androgen is related to HCC, and thus its receptor, AR, also plays an important role [45]. NR1I2 and NR1I3 are related to lipid metabolism and HCC generation [46]. Finally, ESR1 is associated with susceptibility to HCC in HBV carriers [47].

It is a common characteristic that cell proliferation is activated in cancer tissues, thus it would be expected that genes related to the cell cycle are all up-regulated (Module 6). Two regulators, E2F1 and E2F7, were significantly up-regulated (*p*-value < 1e-16), and are well-known TFs in E2F family that controls the cell cycle [48].

To summarize, gene regulation is modular in that each set of regulators regulate specific biological processes. Additionally, the two types of regulators have a clear division of control. We showed that miRNAs control biological functions related to mitochondria and oxidative reduction, while TFs control the immune response, extracellular activities and the cell cycle.

### MiRNA-associated functions in the GRN

To gain a full insight into the functions of miRNAs in the GRN, we performed TAM analysis [49]. The TAM tool takes a list of miRNAs, and returns the enriched functions compared to the whole human miRNAs. Our results for the enriched miRNA-associated functions are listed in Table 2 (FDR < 0.01). As expected, most of the functions are highly related to cancer, such as onco-miRNAs and cell proliferation. Also, we found that functions related to the immune response are enriched for miRNAs. However, according to our analysis of network modules, TFs are mainly responsible for the immune response. From this we inferred that there may be a mechanism by which miRNAs regulate these TFs and further regulate such TF-associated functions indirectly. This concept will be discussed in the following sections in detail. Additionally, we found miRNAs in the GRN are highly enriched in HCC (*p*-value = 5.75e-12, using HMDD [50] as the miRNA category).

**Table 2:** Enriched miRNA-associated functions in the GRN

**Term**	***p*-value**	**FDR**
Human embryonic stem cell (hESC) regulation	8.46e-14	3.64e-12
Inflammation	1.00e-08	2.15e-07
Hematopoiesis	8.00e-08	1.15e-06
Apoptosis	2.70e-07	2.90e-06
Cell cycle-related	4.90e-07	3.81e-06
Hormones regulation	5.60e-07	3.81e-06
Onco-miRNAs	6.20e-07	3.81e-06
Immune response	1.60e-06	8.60e-06
MiRNA tumor suppressors	2.21e-06	1.06e-05
Cell death	4.70e-06	2.02e-05
Cell differentiation	2.40e-05	9.39e-05
Angiogenesis	3.06e-05	1.10e-04
Cell motility	2.34e-04	7.48e-04
Epithelial-mesenchymal transition	2.60e-04	7.48e-04
HIV latency	2.61e-04	7.48e-04
Brain development	2.97e-04	7.99e-04
Chromatin remodeling	3.36e-04	8.31e-04
Immune system	3.48e-04	8.31e-04
Carbohydrate metabolism	1.04e-03	2.35e-03
Akt pathway	1.29e-03	2.78e-03
Bone regeneration	1.72e-03	3.53e-03
Cardiogenesis	3.13e-03	6.12e-03
Cell proliferation	3.95e-03	7.39e-03

### Core gene regulatory network

Although each network module can provide specific control of biological functions, to maintain the integrity of the biology system, dependency exists among modules. Beyond the modularity of gene regulation, there should be a central mechanism to regulate the expression pattern of each module at a higher level. Thus, we introduced the concept of the core GRN that contains the most important regulations among regulators ankd behaviors as a control center for the global GRN.

**Figure 5:**
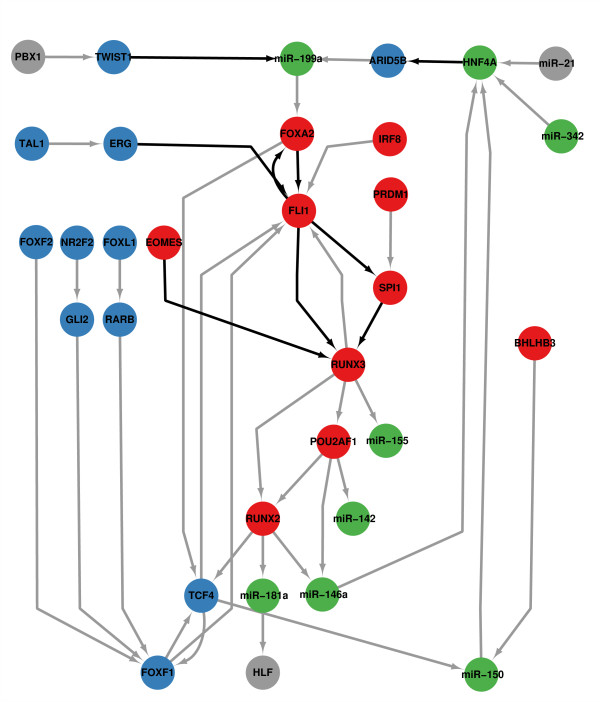
**Core gene regulatory network.** Different colors represent the different modules to which the nodes belong. The color for each module is the same as the color illustrated in Figure 3. Black edges represent the interactions are supported by experiments.

The core GRN is the sub-network extracted from the global GRN, where the nodes in the core GRN are only TFs and miRNAs. Edges in core GRN have the highest edge-betweenness (larger than 99 % quantile) calculated from the global GRN. Edge-betweenness is defined by the number of shortest paths going through an edge in the network, and in the context of GRN, edge-betweenness measures the number of targets that a regulation would affect. In the core GRN, there were 32 nodes and 42 edges. Among them, nine interactions have been supported by previous experiments. In particular, 17 additional experimentally-supported interactions can be inferred from the core GRN indirectly (the list can be found in Additional File 3). The core GRN is illustrated in Figures 3 and 5, and the adjacent list of core GRN can be found in Additional File 4.

The number of edges in the core GRN only covers 1.0 % of all edges in the global GRN, and deletion of these edges does not affect the global GRN's connectivity. Thus, it can be inferred that the local attributes of the network will not be affected by the core GRN. However, the sum of the edge-betweenness takes up 65.8 % of the sum of edge-betweenness in the global GRN. This means that most of the information is controlled by the core GRN, and would affect most of the nodes in the global GRN. When deleting these important edges, the global attributes of the network would be altered and the system would be susceptible to failure.

The core GRN’s role has two aspects. First, it adjusts the regulatory network on the top level. It divides the whole network into two layers with a clear boundary. In the bottom layer, proteins are synthesized under the regulation of TFs and miRNAs, to play roles within or outside of cells. While in the top layer, the core GRN controls what type of proteins would be expressed at what time and at what cellular location. As a result, the entire GRN is organized as a controllable and distributed system. Second, the core GRN can improve the redundancy of the regulatory network. Regulators and regulatory relationships in the core GRN can control more than one module, and regulations of the non-regulator proteins are influenced by the core GRN through a variety of paths. Therefore, when a regulation path does not work for some proteins, the system will assign other paths to process regulations in order to avoid the overall collapse caused by a small portion of damage. In addition, a large number of feedforward and feedback loops exist in the core GRN, which contribute to the flexibility, resiliency and stability of the core GRN, and further to the stability of the whole regulatory network.

In the core GRN, most of the regulators are related to cancers. PBX1 [51], TWIST1 [52], HNF4A [53], ERG [54], FOXA2 [55], NR2F2 [56], FLI1 [57], GLI2 [58], RARB [59], RUNX3 [30], BHLHB3 [60], RUNX2 [61], TCF4 [36] and FOXF1 [62] are reported TFs related to cancers. After querying the human microRNA disease database (HMDD) [50], we found miR-21 [63], miR-199a [42], miR-155 [64], miR-142 [65], miR-181a [66], miR-146a [41], and miR-150 [40] are reported miRNAs related to cancers. Especially, there is direct evidence for the involvement of TWIST1, HNF4A, GLI2, RARB, RUNX3, TCF4, FOXF1, miR-21, miR-199a, miR-155, miR-142, miR-146a, miR-181a, and miR-150 in HCC generation.

### Transcription-level regulation of biological pathways

**Figure 6:**
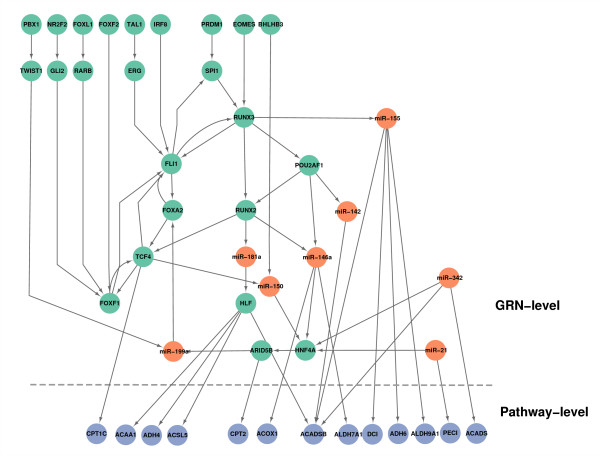
**Gene regulation of the fatty acid metabolism pathway by the core GRN.** Nodes in green represent TFs; Nodes in orange represent miRNAs; Nodes in blue represent genes in pathways.

In the complete cellular system, there exist several kinds of biological networks: metabolic networks containing chemical reactions between metabolites and enzymes, protein-protein interaction networks containing protein modification and signaling transduction, and the gene regulatory network. The aim of GRN control is to regulate the quantity of downstream proteins, and to further influence the protein-protein interaction and metabolic networks. For a type of specific biological network, pathways are a set of genes and molecules that act together in the form of both metabolic and protein-protein interactions to carry out certain biological functions. It may explain how pathways are affected in diseases from the viewpoint of gene regulation of pathways. Thus, we predicted the regulations of KEGG pathways by the GRN. We found enriched pathways from all genes in the GRN, and the significant pathways are listed in Table 3 (FDR < 0.05). Most of the enriched pathways are highly related to HCC, such as fatty acid metabolism, which is associated with tumors [67] and cell adhesion. An example of the regulation of the fatty acid metabolism pathway is illustrated in Figure 6, where the top part is the GRN level and the bottom part is the pathway level. It may provide insights to explain how fatty acid metabolism is altered under the control of the GRN. For regulations of all significant pathways by core the GRN, readers can refer to Additional File 5.

**Table 3:** Enriched KEGG pathways of genes in the GRN

**KEGG pathway**	***p*-value**	**FDR**
hsa00071:Fatty acid metabolism	1.04e-06	1.93e-04
hsa04660:T cell receptor signaling pathway	1.69e-06	1.56e-04
hsa04514:Cell adhesion molecules (CAMs)	7.74e-06	4.77e-04
hsa04640:Hematopoietic cell lineage	1.25e-04	5.75e-03
hsa04512:ECM-receptor interaction	2.42e-04	8.91e-03
hsa04610:Complement and coagulation cascades	3.18e-04	9.76e-03
hsa03320:PPAR signaling pathway	3.18e-04	9.76e-03
hsa00280:Valine, leucine and isoleucine degradation	3.90e-04	1.03e-02
hsa05340:Primary immunodeficiency	4.71e-04	1.08e-02
hsa00620:Pyruvate metabolism	4.98e-04	1.02e-02
hsa04510:Focal adhesion	6.56e-04	1.21e-02
hsa00830:Retinol metabolism	1.21e-03	2.02e-02
hsa04666:Fc gamma R-mediated phagocytosis	1.48e-03	2.26e-02
hsa00640:Propanoate metabolism	3.06e-03	4.27e-02

## Conclusions

In this study, we established the gene regulatory network that is involved in HCC generation. We revealed that the GRN is modular, where different sets of regulators take charge of specific biological functions. Among them, miRNAs mainly regulate mitochondria and oxidative reduction, while TFs regulate the immune responses, extracellular activity and the cell cycle. At a higher level, a core GRN exists to organize regulations among different modules and to maintain the robustness of the whole network.

Our strategy for constructing the GRN has two advantages. First, the interactions were integrated from three data sources, which are target prediction, experimentally-supported interactions and microarray data. Target prediction provides potential evidence for direct physical interactions, while microarray data can measure the correlations between regulators and targets. Moreover, experimentally-supported interactions can help to improve the quality of the network. Three data sources assign relations to regulators and targets from different aspects, and the integration of the three data sources can make the result more reliable. Second, cutoff of correlations for TFs and miRNAs were set independently of one another. We found that the regulation strength differed from TFs to miRNAs, and the reason for this unequal strength is probably due to the mechanisms of the two kinds of regulators. The selection of cutoffs was processed by a scale-free topological criterion to ensure the final network is biologically sensible.

HCC is a complex disease that involves various molecule interactions. Analysis of the gene regulatory network can help to reveal the mechanisms involved in the development of HCC. In this article we applied a strategy to reveal the most important regulations at the transcriptional level and post-transcriptional level from a systematic view. The core gene regulatory network proposed is highly related to HCC, and we believe it will provide valuable insights for further experimental validations.

## Methods

### Processing of microarray data

Microarray data for liver cancer was downloaded from the GEO database [GEO: GSE22058]. The experiment examined genome-wide expression profiles of both miRNAs and mRNAs from paired tumor (TU) and adjacent non-tumor tissues (AN) from a cohort of 96 HCC patients. All HCC patients were infected by HBV. We utilized intensity values that have been normalized by RMA algorithm, and combined the intensities of tumor and adjacent non-tumor tissue into ratio values. To avoid the occurrence of the division of small intensities leading to high ratio values, we added a penalized term *α* to calculate the ratio, as in Formula 1, where *α* is the mean value of 25 % quantile of intensity in tumor and adjacent non-tumor respectively.

(1) $$\begin{array}{l} ratio=\log\left(\frac{{\text{intensity}}_{\mathrm{TU}}+\alpha }{{\text{intensity}}_{\mathrm{AN}}+\alpha }\right)\\ \alpha =\frac{q_{0.25}({\text{intensity}}_{\mathrm{TU}})+q_{0.25}({\text{intensity}}_{\mathrm{AN}})}{2} \end{array}$$

Since there are multiple probes for some genes, the ratio values for genes were merged by averaging the ratio of their probes. After merging multiple probes, in total there were 18503 genes and 202 miRNAs. These genes and miRNAs were taken as the candidate list in which we looked for gene regulations.

### Target prediction

Promoters of genes and miRNAs were retrieved from UCSC genome browser (hg19) [68], and the upstream 5 kb sequences were taken. We used TFs identified in the TRANSFAC database [69] and utilized MATCH [70] to predict regulations of genes by TFs and miRNAs by TFs. MATCH was executed using the default settings. Regulations of genes by miRNAs were predicted by TargetScan 6.0 [71]. We first obtained miRNA lists containing broadly conserved miRNA families, conserved miRNA families and poorly conserved miRNA families, and then retrieved web pages of conserved targets for each miRNA family using the TargetScan on-line search tool. We analyzed web pages with details of miRNA targets, and noted all the interactions listed. In the target prediction procedure, after mapping to existing gene lists and miRNA lists obtained through microarrays, we identified 1450405 TF regulations of genes, 27077 TF regulations of miRNAs and 277392 miRNAs regulations of genes. We did not use species conservation information in the TF target prediction procedure and did not make any restriction on miRNA targets predictions because some regulatory steps are not conserved and the use of the species conservation would miss these positive interactions.

### Experimentally-supported interactions

Experimentally-supported regulations of targets by TFs were downloaded from the ChEA manual database (a collection of TF-to-gene regulations from ChIP-chip and ChIP-seq publications) [19]. Regulations from miRNAs to targets were downloaded from TarBase 6.0 [20]. Regulations from TFs to miRNAs were downloaded from TransmiR 1.1 [21]. After mapping to existing gene lists and miRNA lists obtained through microarrays, there were 85025 interactions from TFs to genes, 15500 interactions from miRNAs to genes and 202 interactions from TFs to miRNAs that have been experimentally validated.

### Co-expression model

We only considered correlations between regulators and targets that have predicted target interactions. We assumed the expression vector for a certain regulator as *y* and the expression vector for one of its targets as *x*. First univariate linear regression was applied to *y* and *x*, and then Cook’s distance was calculated to estimate the validity of the data points. We set data points as outliers where Cook’s distance were larger than 0.5. The outliers were then eliminated, and the Pearson correlation coefficient between regulator and target was calculated.

### Topological criterion

We denoted the node degree as *k*. For one specific form of network, *k* follows a power-law distribution [26] (Formula 2). Networks with such attributes are well-known as scale-free networks. In these networks, a minority of nodes dominates most of the connections; however in the real world, a lot of biological networks such as protein-protein interactions, metabolism networks and transcriptional regulatory networks are thought to be scale-free. Thus we assumed scale-free is an important attribute for large biological networks. Under some conditions when the size of the network is moderate or even small, the degree distribution would be shifted to an exponential truncated power-law distribution [27] (formula 3). For the construction of GRNs from the co-expression model, different cutoffs of absolute value of correlation coefficients would result in different networks. The cutoff was selected under the criterion that the final network is approximately scale-free, which is measured by the degree distribution. The real degree distribution in the GRN is fitted to the power-law distribution or the exponential truncated power-law distribution, and the *R*^2^ value is used to measure the goodness-of-fit. Since the GRN is a directed network, the distribution of degree is divided into in-degree distribution and out-degree distribution. In Formulas 2 and 3, *γλ* and *α* are network parameters.

(2) $$P(k)∼k^{-\gamma }$$

(3) $$P(k)∼k^{-\lambda }e^{-\alpha k}$$

### Functional Enrichment

Functional enrichment was applied to evaluate whether a group of genes share common biological functions. We used DAVID [72] to perform Gene Ontology enrichment and pathway enrichment. For Gene Ontology enrichment of genes in each module, we selected GOTERM_BP_FAT, GOTERM_CC_FAT and GOTERM_MF_FAT as the gene set categories. For pathway enrichment of whole genes in the GRN, KEGG was selected as the gene set category.

Functional enrichment for miRNAs was applied by TAM [49] to evaluate whether a group of miRNAs regulate common functions or are involved in common diseases. We used miRNAs in the GRN as the candidate miRNA list, and looked for over-represented functions under the category of ‘miRNA function’. We selected all miRNAs in TAM as background, and the analysis was performed under miRNA set version 2. Since we did not take all miRNA categories implemented in TAM, the false discovery rates (FDRs) were re-calculated using BH method [73].

### Network analysis

The igraph package (version 0.5.5) [74] in R statistical environment was utilized to analyze networks. Cytoscape (version 2.8.2) was used to visualize the network [75].

## Abbreviations

HCC, hepatocellular carcinoma; TF, transcription factor; GRN, gene regulatory network; HMDD, human microRNA disease database; FDR, false discovery rate.

## Competing interests

The authors declare that they have no competing interests.

## Authors’ contributions

ZG performed the analysis and wrote the manuscript. JW and CZ conceived the study, and helped to draft the manuscript. All authors have read and approved the final manuscript.

## Supplementary Material

Additional file 1 The gene regulatory network in adjacency list format. Click here for file

Additional file 2 The core gene regulatory network in adjacency list format. Click here for file

Additional file 3: Experimentally-supported interactions in the core GRN. Click here for file

Additional file 4: Gene Ontology enrichment for genes in six modules of the GRN. Click here for file

Additional file 5: Regulations of the enriched KEGG pathways by the core GRN. Click here for file
