# Supplementary material for: Gene regulation is governed by a core network in hepatocellular carcinoma
Source: BMC Syst Biol. 2012 May 1;6:32. doi: 10.1186/1752-0509-6-32 (PMC3403900; doi:10.1186/1752-0509-6-32)
Supplement: Additional file 4: — Gene Ontology enrichment for genes in six modules of the GRN. [file 1752-0509-6-32-S4.gz › enrichment-of-modules/index.html]

Gene Ontology enrichment for genes in six largest modules in GRN


#### Gene Ontology enrichment for genes in six largest modules in GRN

| Module index | Target gene functions |
| --- | --- |
| 1 | Immune response, Plasma membrane, Leukucyte cell activation |
| 2 | Extracellular region,  Cell adhension |
| 3 | Mitochondrion,  Oxidative reduction,  Mitochondrial envelope |
| 4 | Oxidative reduction,  Cofactor metabolic process, Steroid metabolic process |
| 5 | Oxidative reduction, Microsome,  Fatty acid metabolic process |
| 6 | Cell cycle, Mitosis, Chromosome,  Nuclear lumen |
| 3, 4, 5 | Mitochondrion, Oxidative reduction, Cofactor binding |

First column in the table corresponds to module index in article. In each heatmap, rows represent Gene Ontology terms, columns represent genes. Cells with blue color means there're genes in the corresponding Gene Ontology terms. The red column represents p-values for Gene Ontology terms after enrichment. Only Gene Ontology terms with false discovery rate (FDR) less than 0.05 are illustrated in figures. If the number of Gene Ontology terms is larger than 20, the top 20 most significant Gene Ontology terms are illustrated.
