# Supplementary figures and images for: Gene regulation is governed by a core network in hepatocellular carcinoma
Source: BMC Syst Biol. 2012 May 1;6:32. doi: 10.1186/1752-0509-6-32 (PMC3403900; doi:10.1186/1752-0509-6-32)

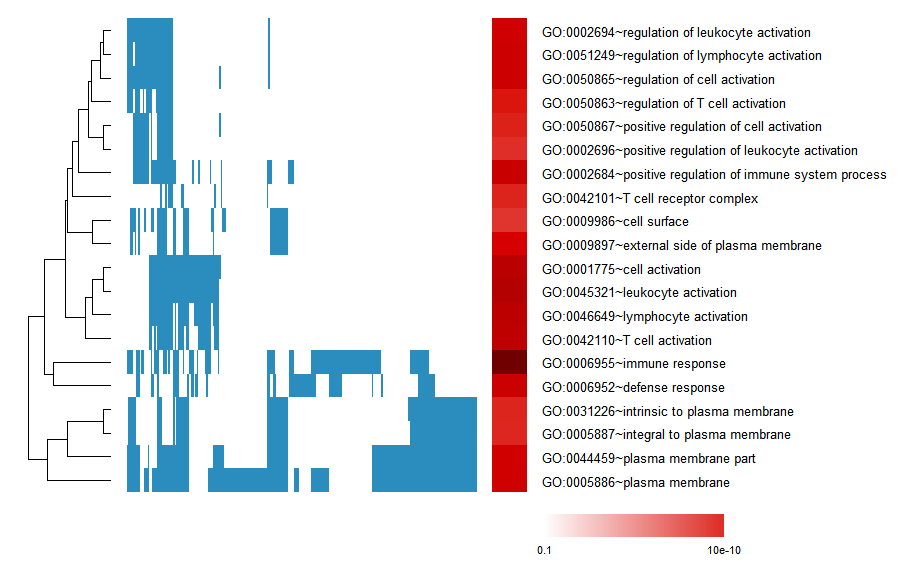

Supplement: Additional file 4: — Gene Ontology enrichment for genes in six modules of the GRN. [file 1752-0509-6-32-S4.gz › enrichment-of-modules/m1.png]

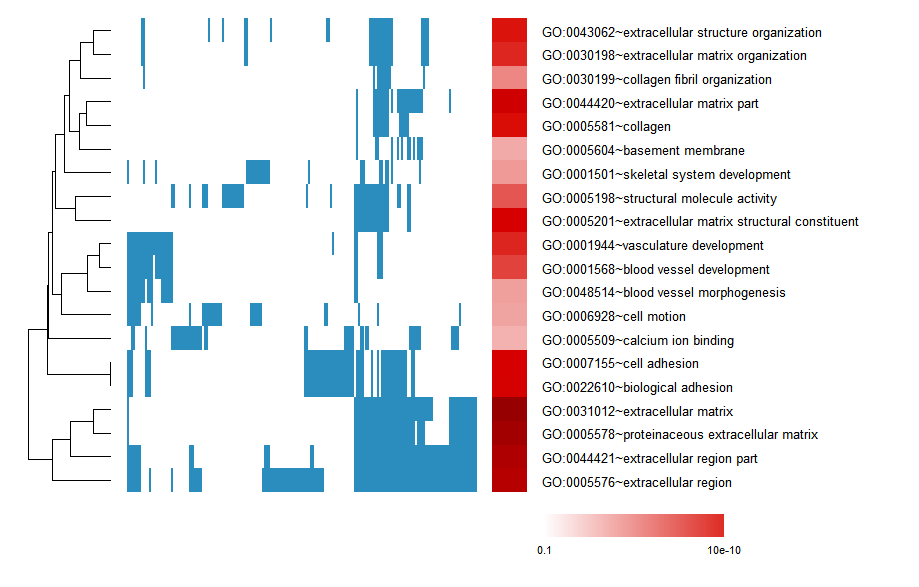

Supplement: Additional file 4: — Gene Ontology enrichment for genes in six modules of the GRN. [file 1752-0509-6-32-S4.gz › enrichment-of-modules/m2.png]

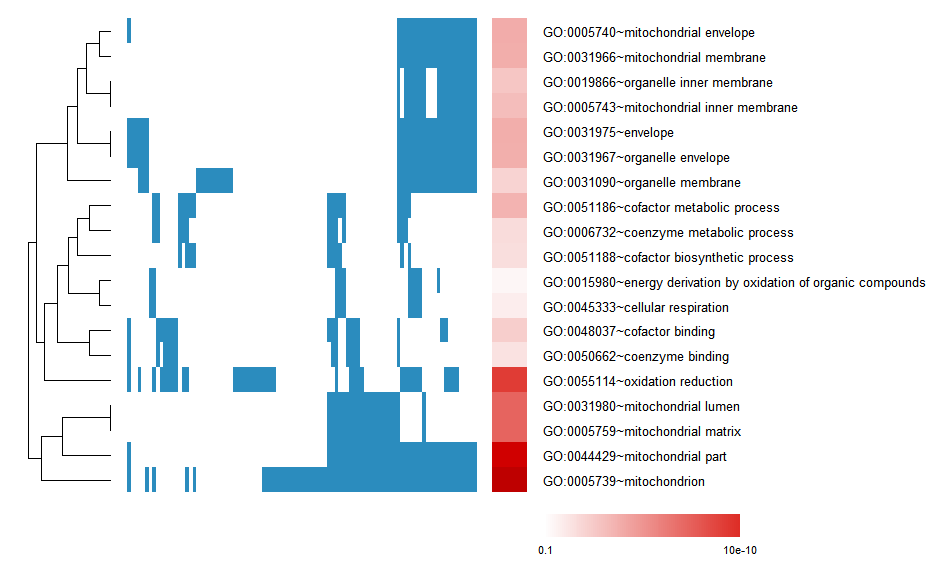

Supplement: Additional file 4: — Gene Ontology enrichment for genes in six modules of the GRN. [file 1752-0509-6-32-S4.gz › enrichment-of-modules/m3.png]

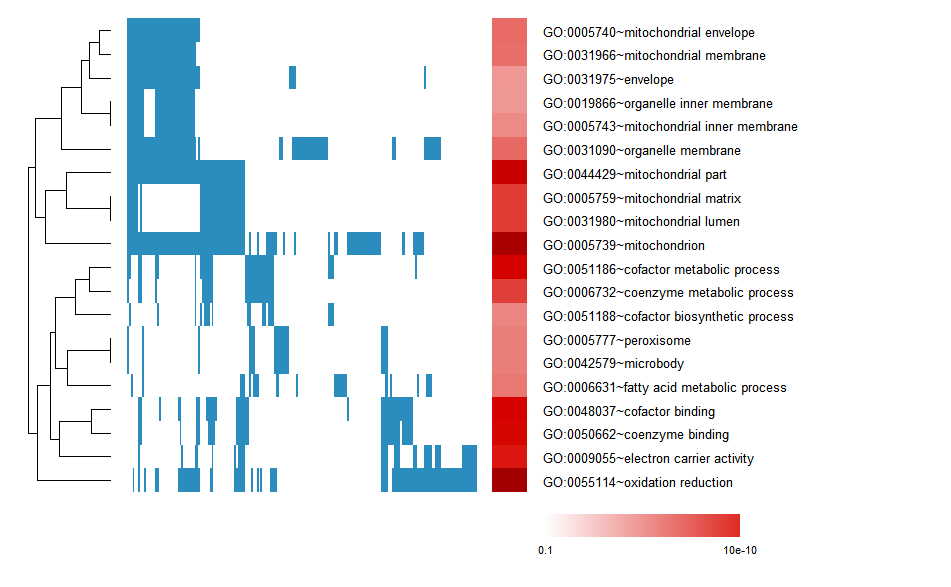

Supplement: Additional file 4: — Gene Ontology enrichment for genes in six modules of the GRN. [file 1752-0509-6-32-S4.gz › enrichment-of-modules/m345.png]

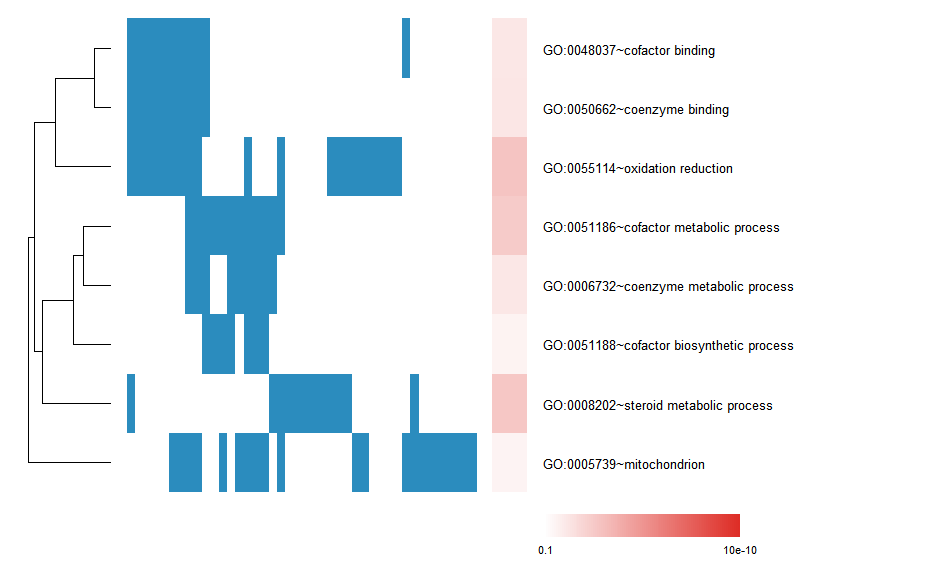

Supplement: Additional file 4: — Gene Ontology enrichment for genes in six modules of the GRN. [file 1752-0509-6-32-S4.gz › enrichment-of-modules/m4.png]

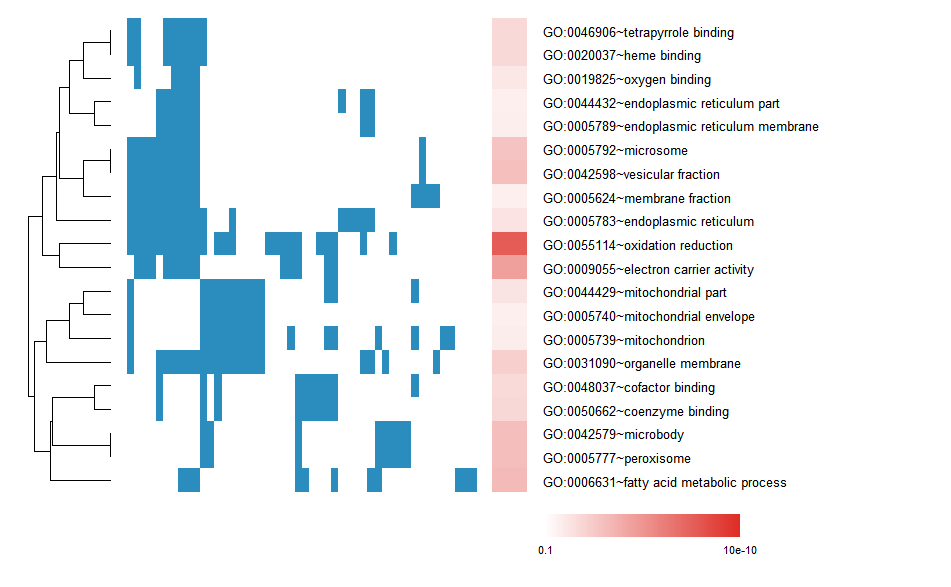

Supplement: Additional file 4: — Gene Ontology enrichment for genes in six modules of the GRN. [file 1752-0509-6-32-S4.gz › enrichment-of-modules/m5.png]

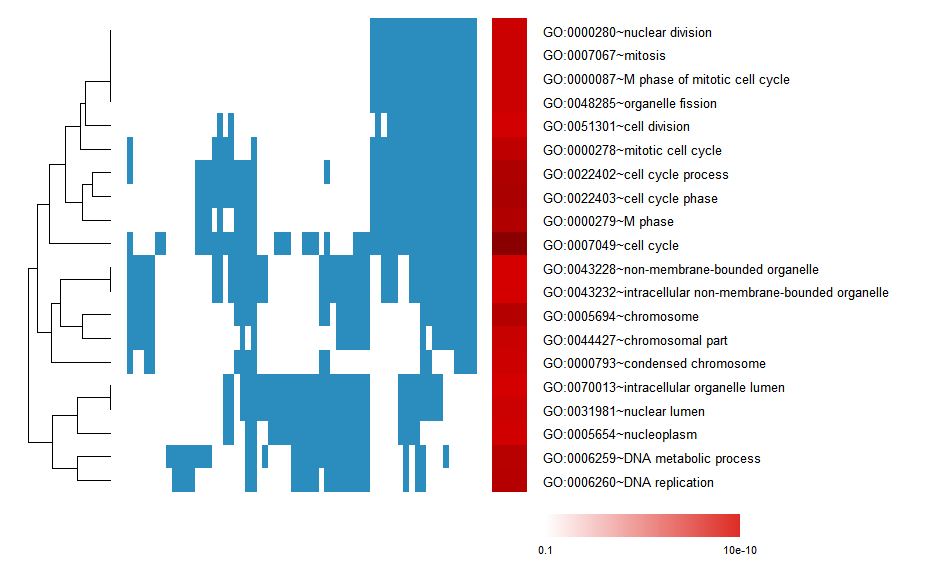

Supplement: Additional file 4: — Gene Ontology enrichment for genes in six modules of the GRN. [file 1752-0509-6-32-S4.gz › enrichment-of-modules/m6.png]

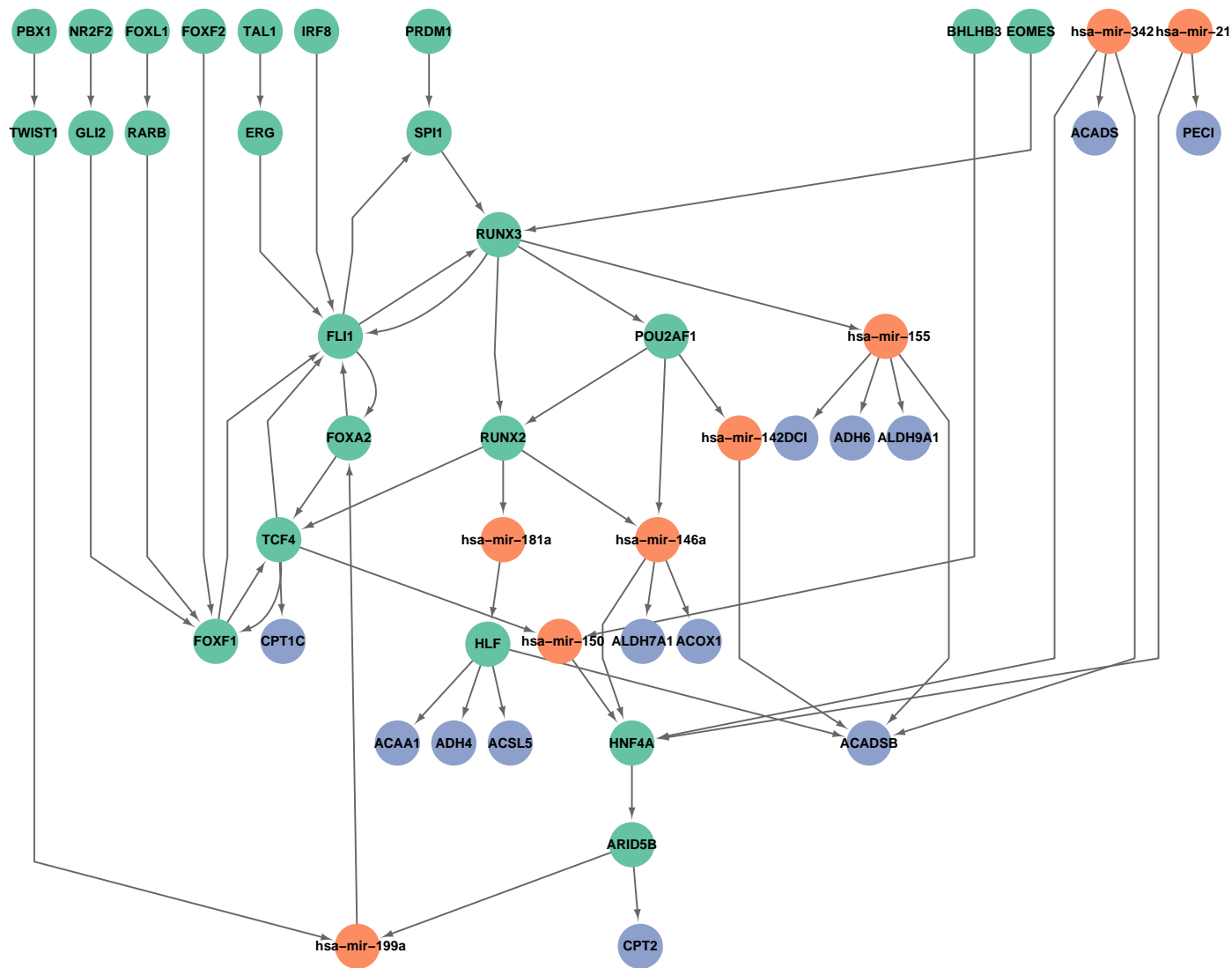

Supplement: Additional file 5: — Regulations of the enriched KEGG pathways by the core GRN. [file 1752-0509-6-32-S5.gz › regulation-on-pathway/path1.pdf]

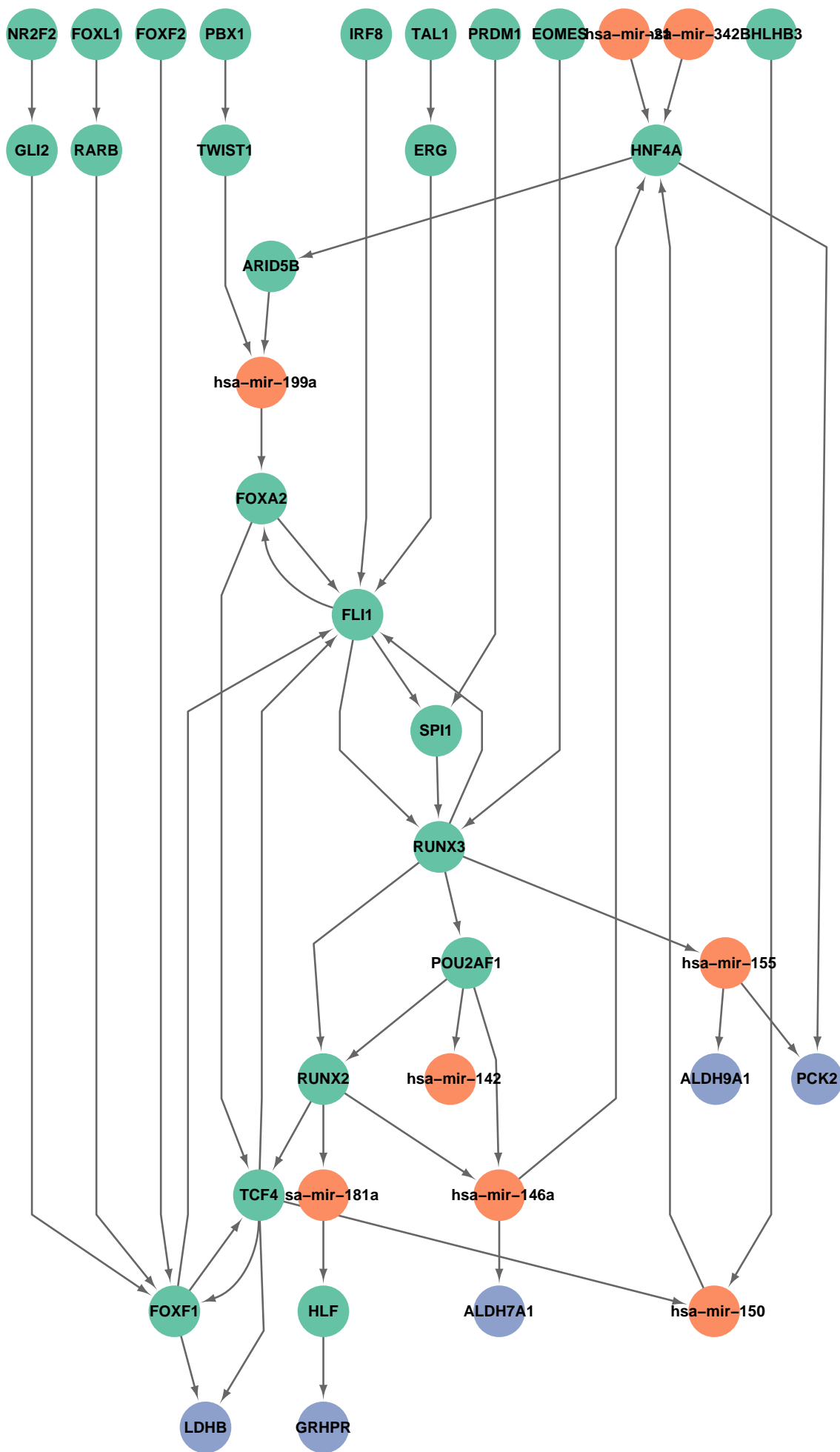

Supplement: Additional file 5: — Regulations of the enriched KEGG pathways by the core GRN. [file 1752-0509-6-32-S5.gz › regulation-on-pathway/path10.pdf]

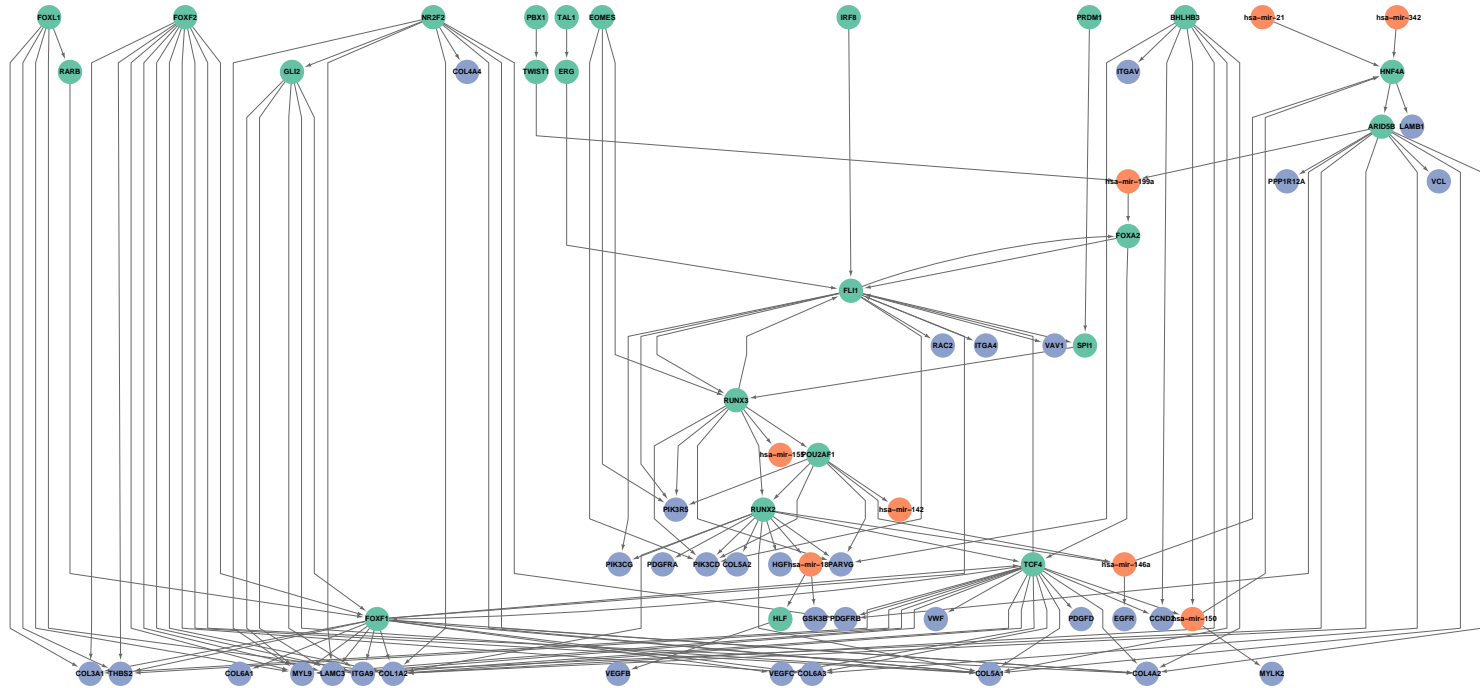

Supplement: Additional file 5: — Regulations of the enriched KEGG pathways by the core GRN. [file 1752-0509-6-32-S5.gz › regulation-on-pathway/path11.pdf]

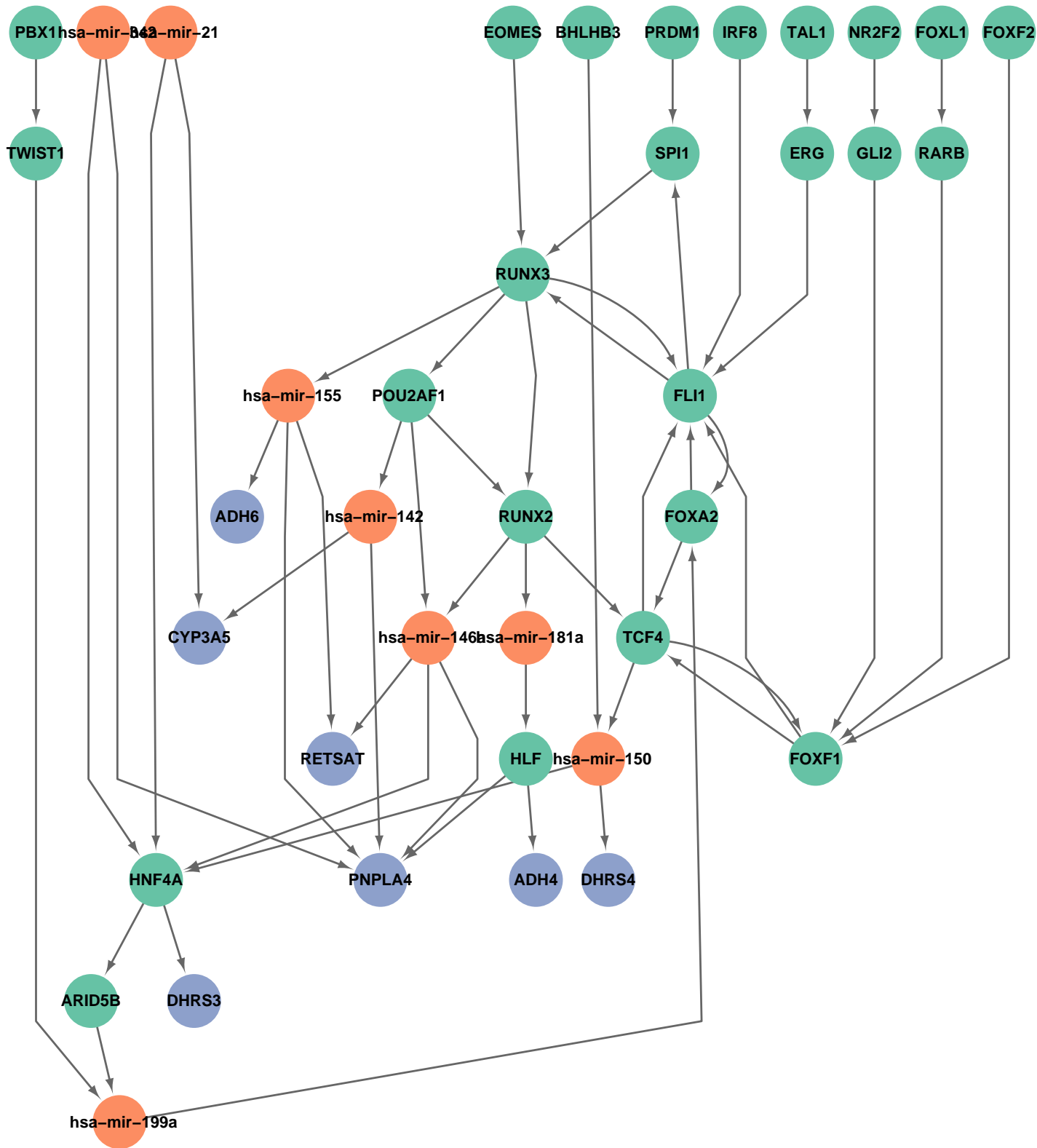

Supplement: Additional file 5: — Regulations of the enriched KEGG pathways by the core GRN. [file 1752-0509-6-32-S5.gz › regulation-on-pathway/path12.pdf]

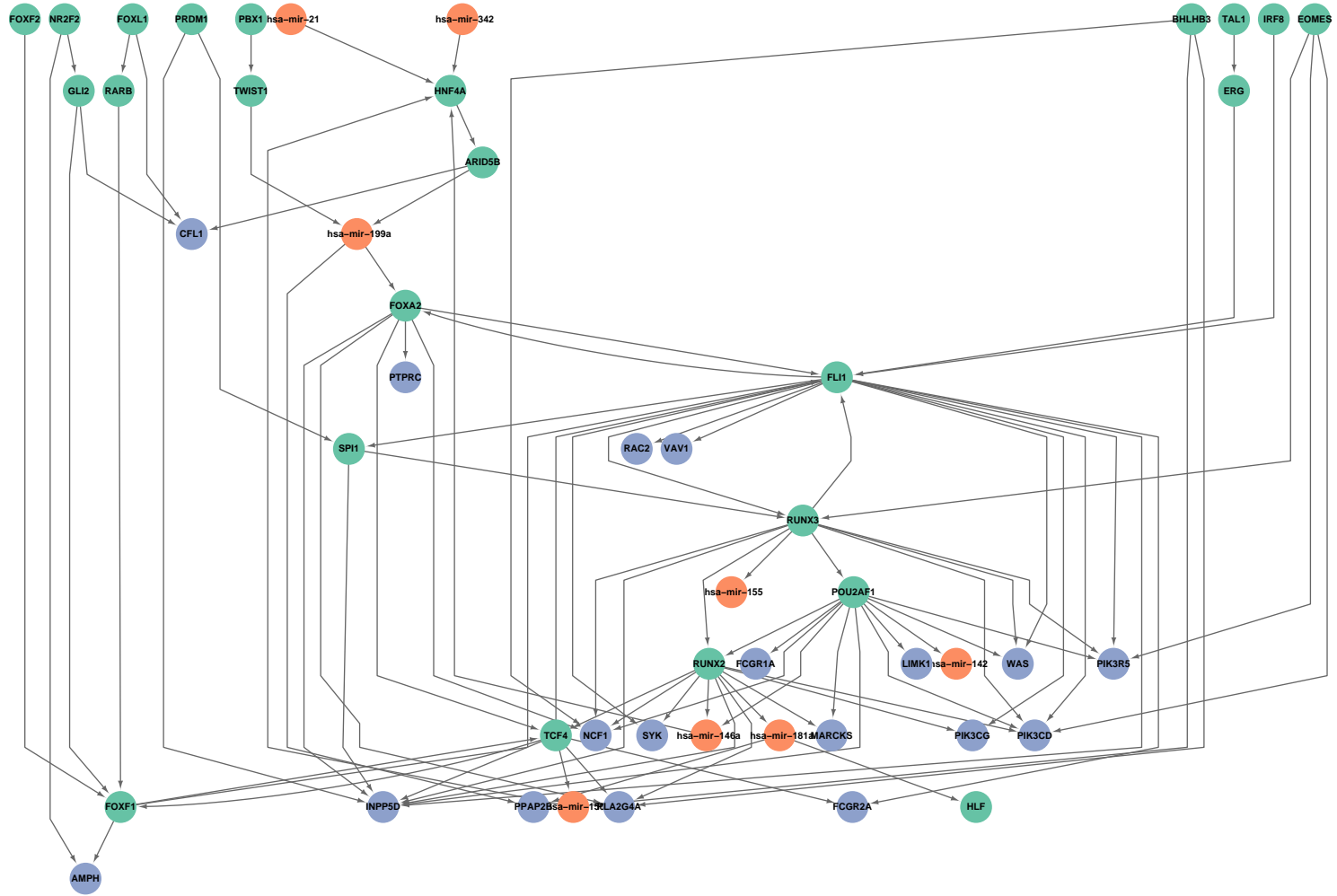

Supplement: Additional file 5: — Regulations of the enriched KEGG pathways by the core GRN. [file 1752-0509-6-32-S5.gz › regulation-on-pathway/path13.pdf]

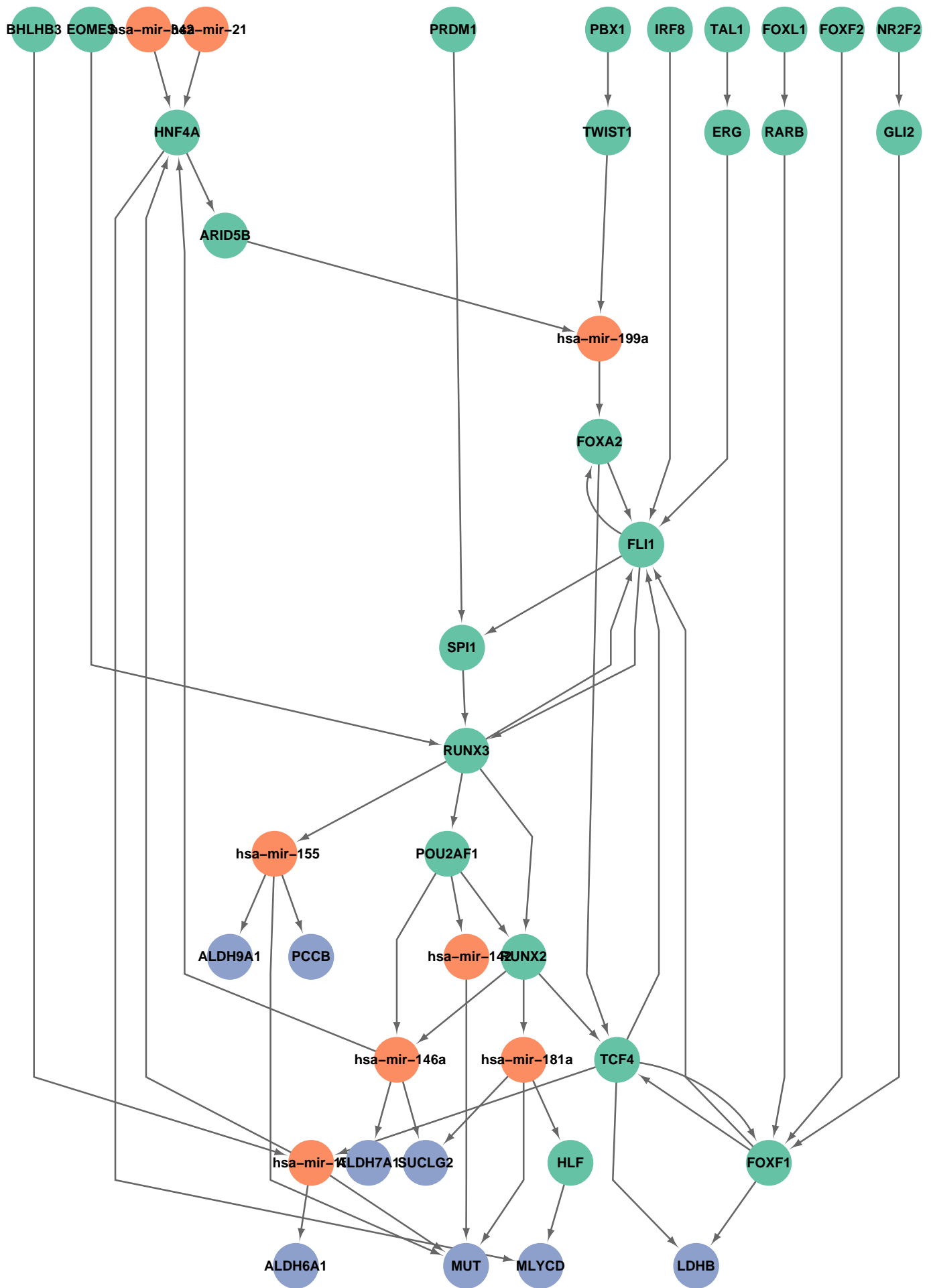

Supplement: Additional file 5: — Regulations of the enriched KEGG pathways by the core GRN. [file 1752-0509-6-32-S5.gz › regulation-on-pathway/path14.pdf]

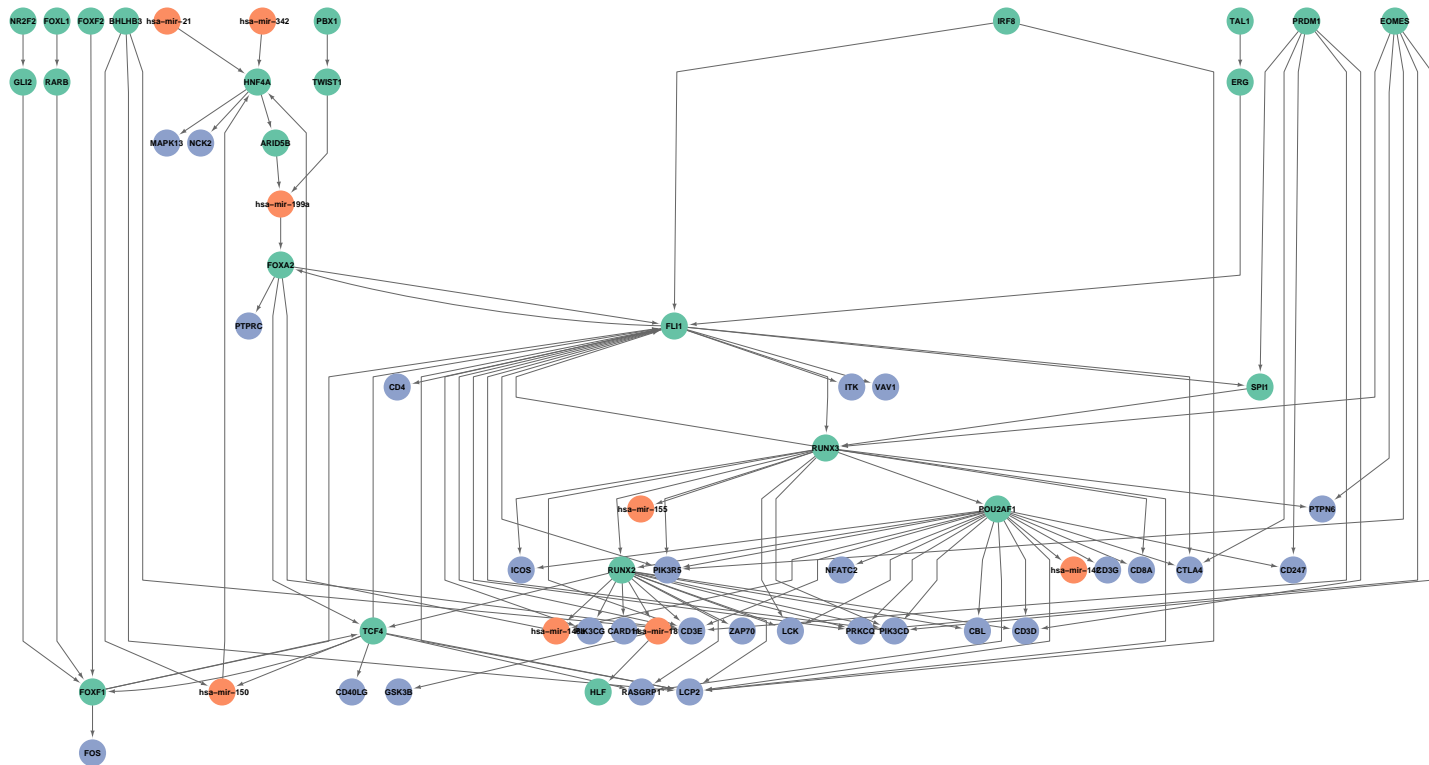

Supplement: Additional file 5: — Regulations of the enriched KEGG pathways by the core GRN. [file 1752-0509-6-32-S5.gz › regulation-on-pathway/path2.pdf]

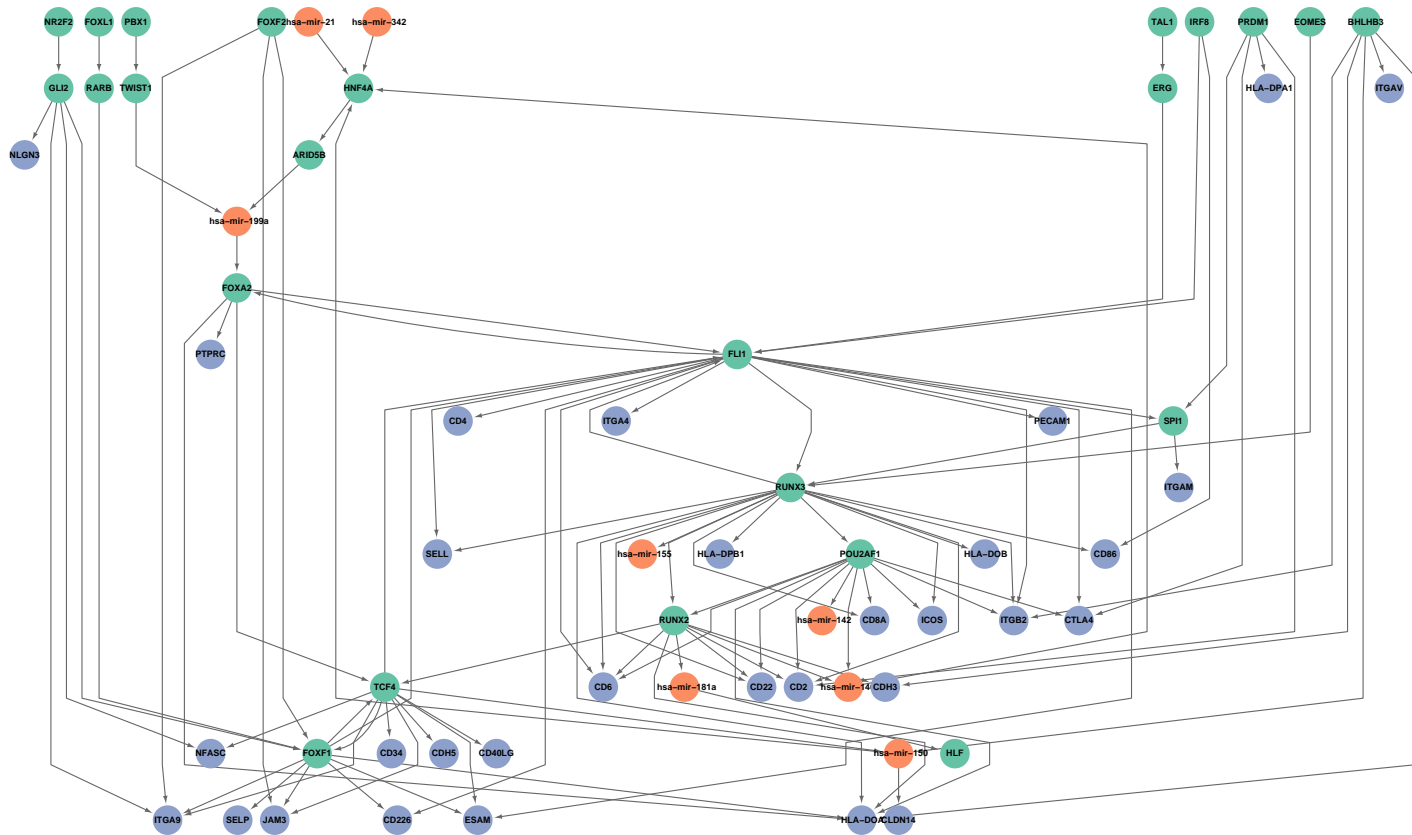

Supplement: Additional file 5: — Regulations of the enriched KEGG pathways by the core GRN. [file 1752-0509-6-32-S5.gz › regulation-on-pathway/path3.pdf]

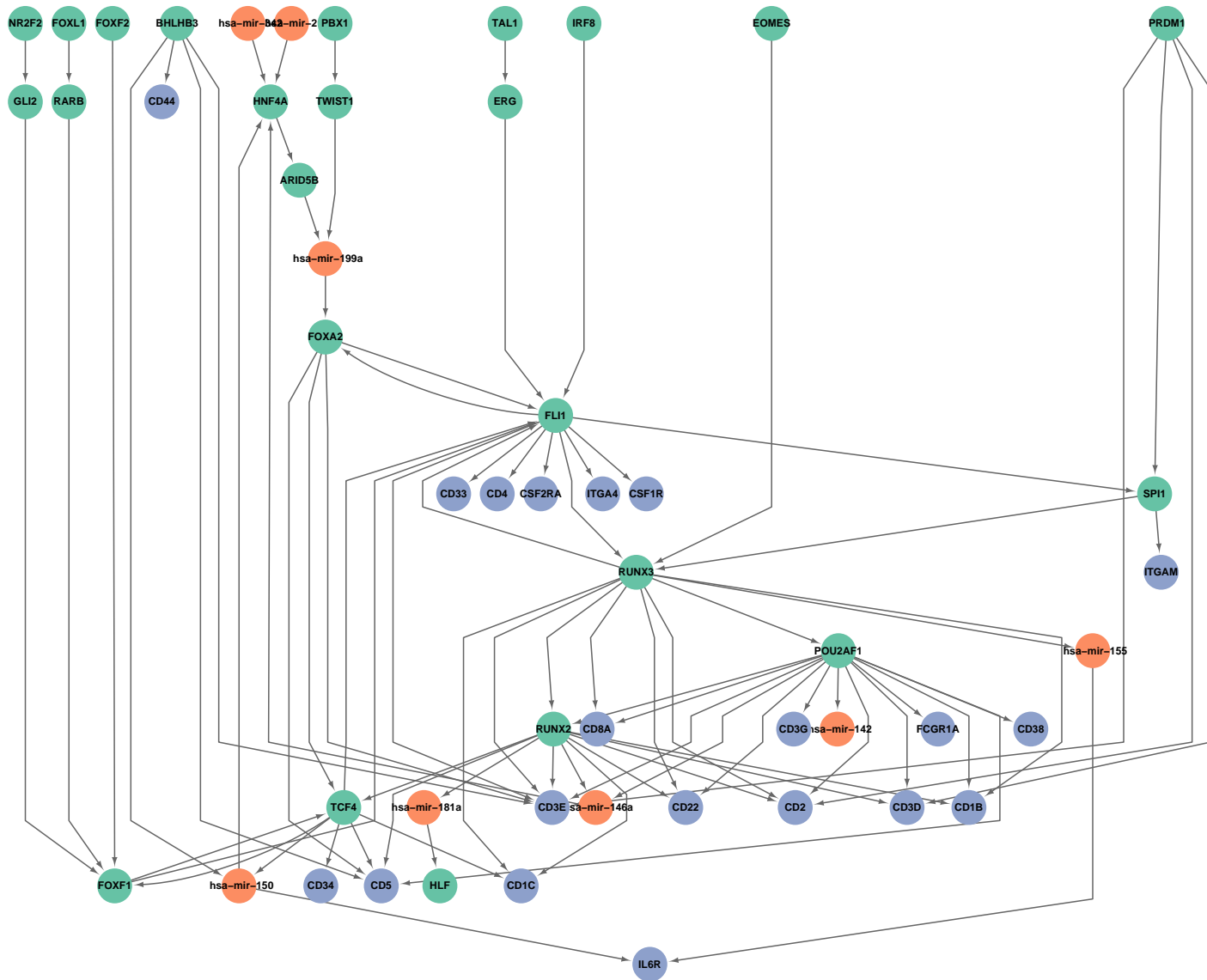

Supplement: Additional file 5: — Regulations of the enriched KEGG pathways by the core GRN. [file 1752-0509-6-32-S5.gz › regulation-on-pathway/path4.pdf]

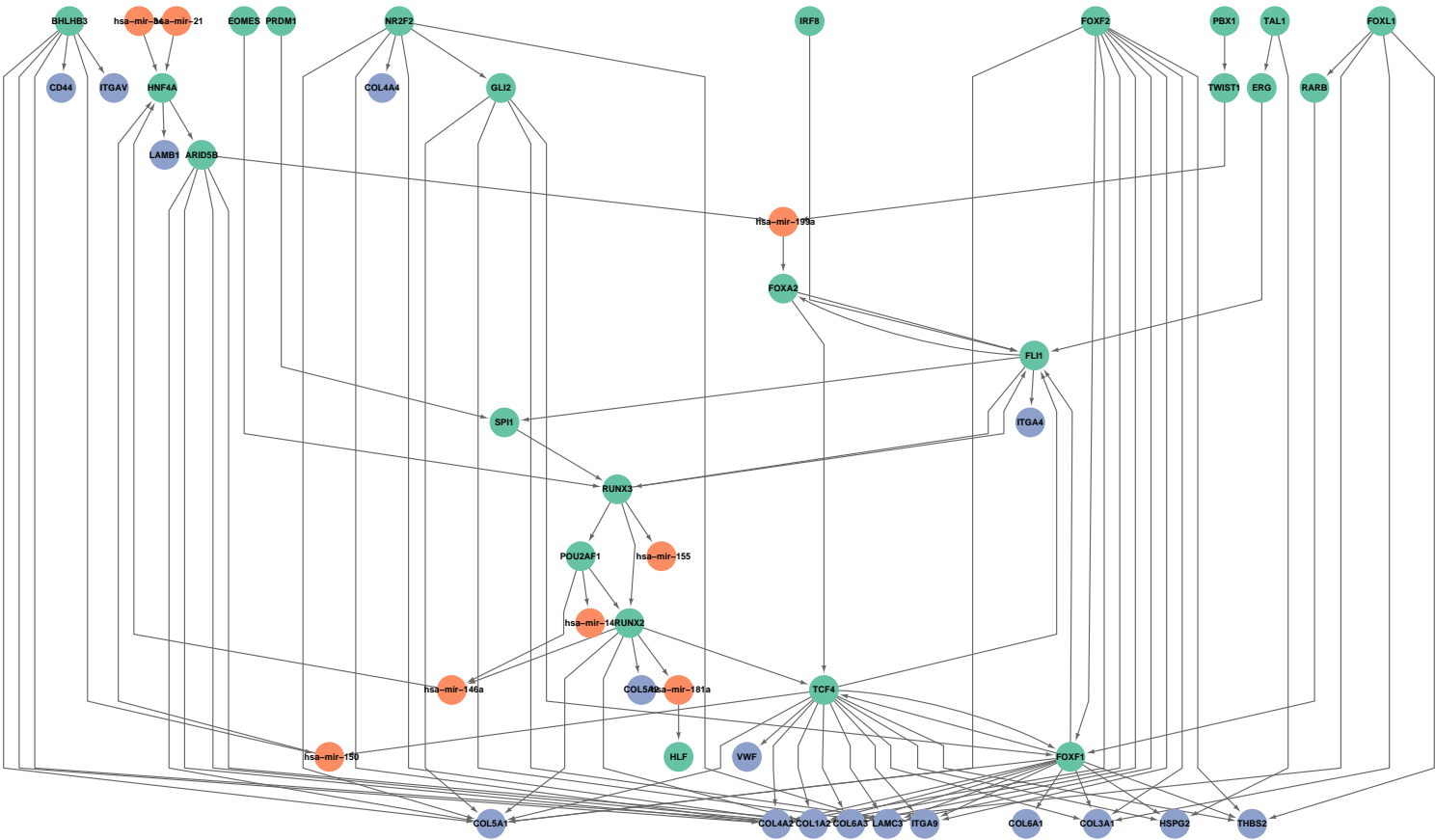

Supplement: Additional file 5: — Regulations of the enriched KEGG pathways by the core GRN. [file 1752-0509-6-32-S5.gz › regulation-on-pathway/path5.pdf]

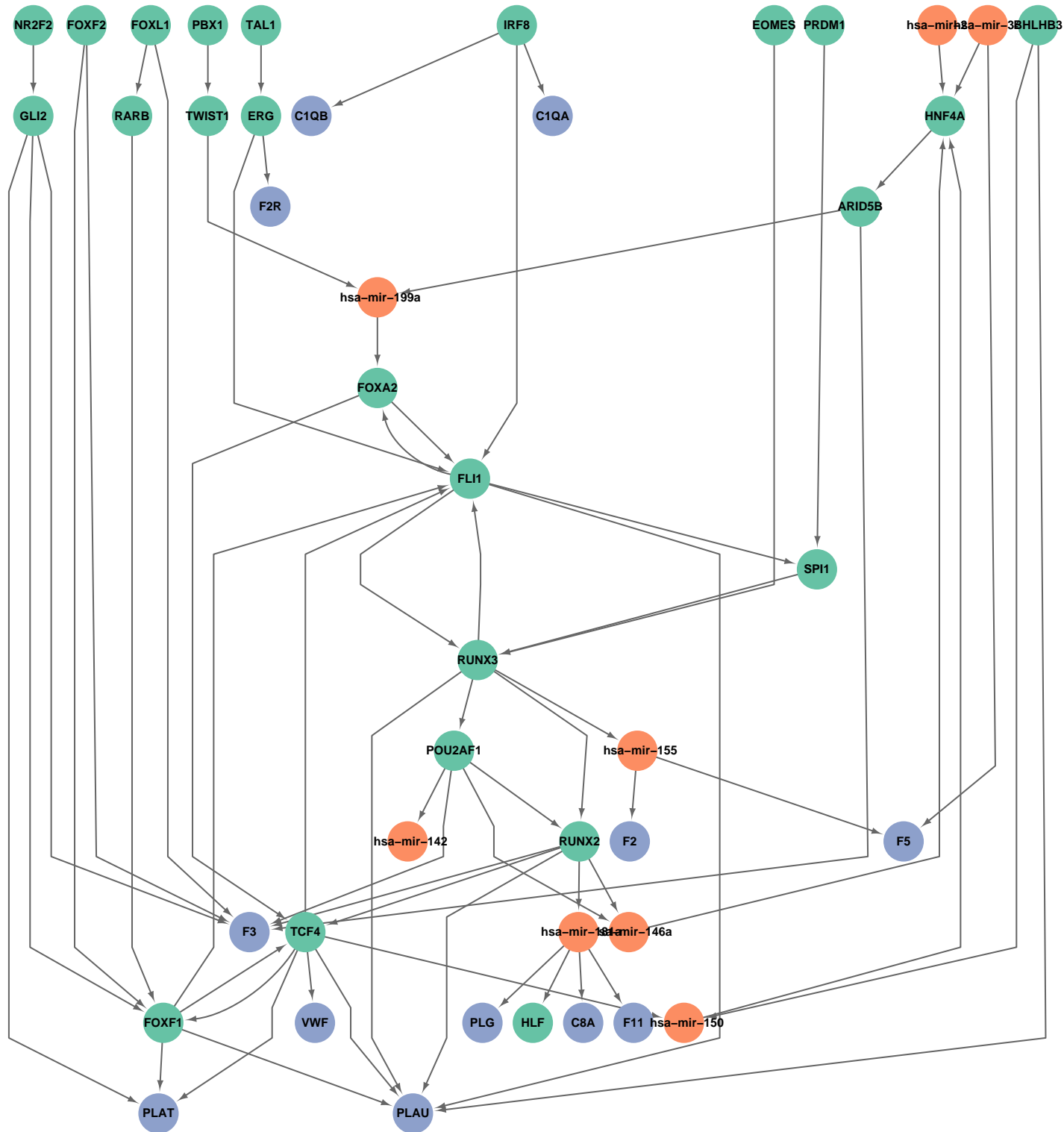

Supplement: Additional file 5: — Regulations of the enriched KEGG pathways by the core GRN. [file 1752-0509-6-32-S5.gz › regulation-on-pathway/path6.pdf]

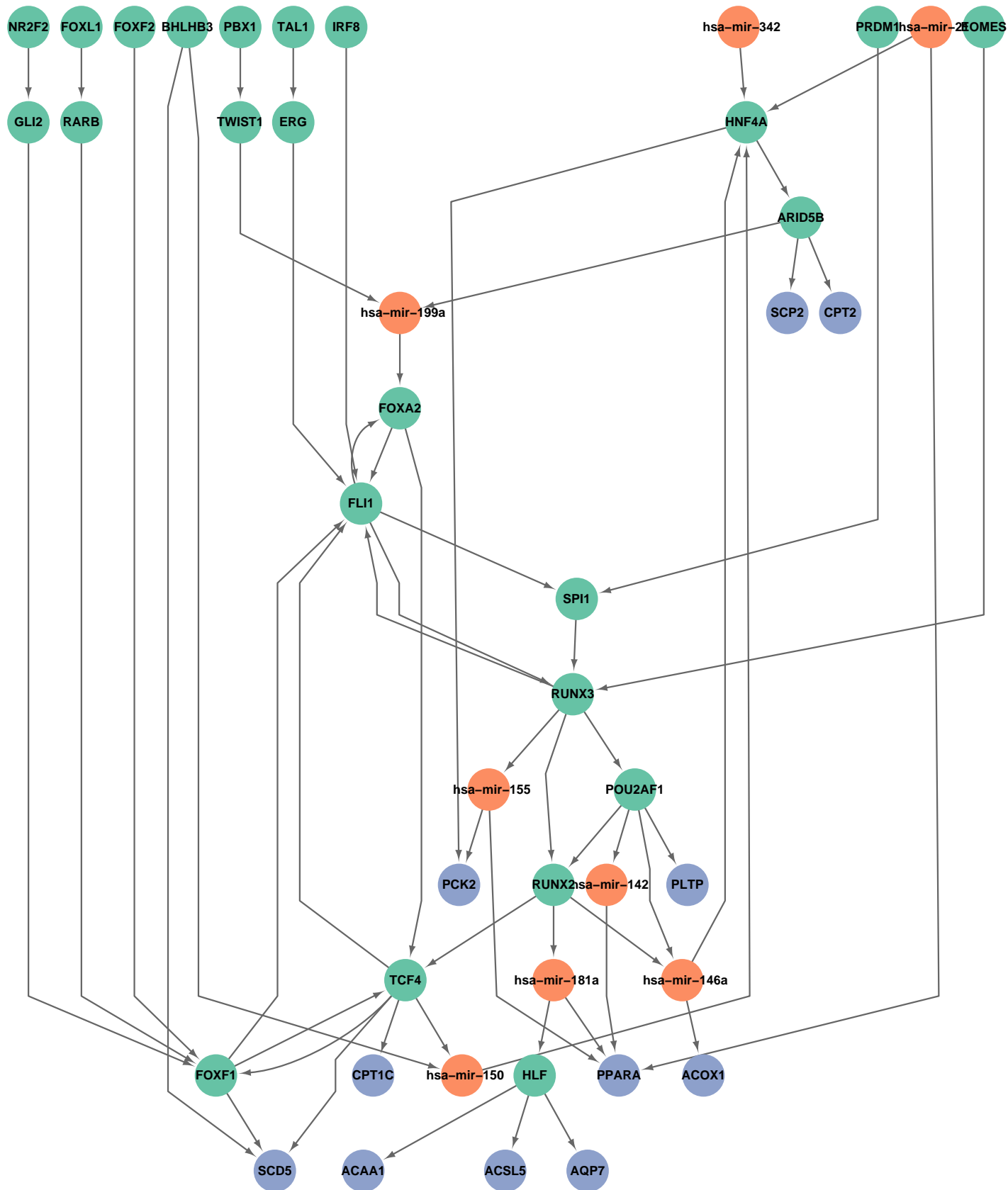

Supplement: Additional file 5: — Regulations of the enriched KEGG pathways by the core GRN. [file 1752-0509-6-32-S5.gz › regulation-on-pathway/path7.pdf]

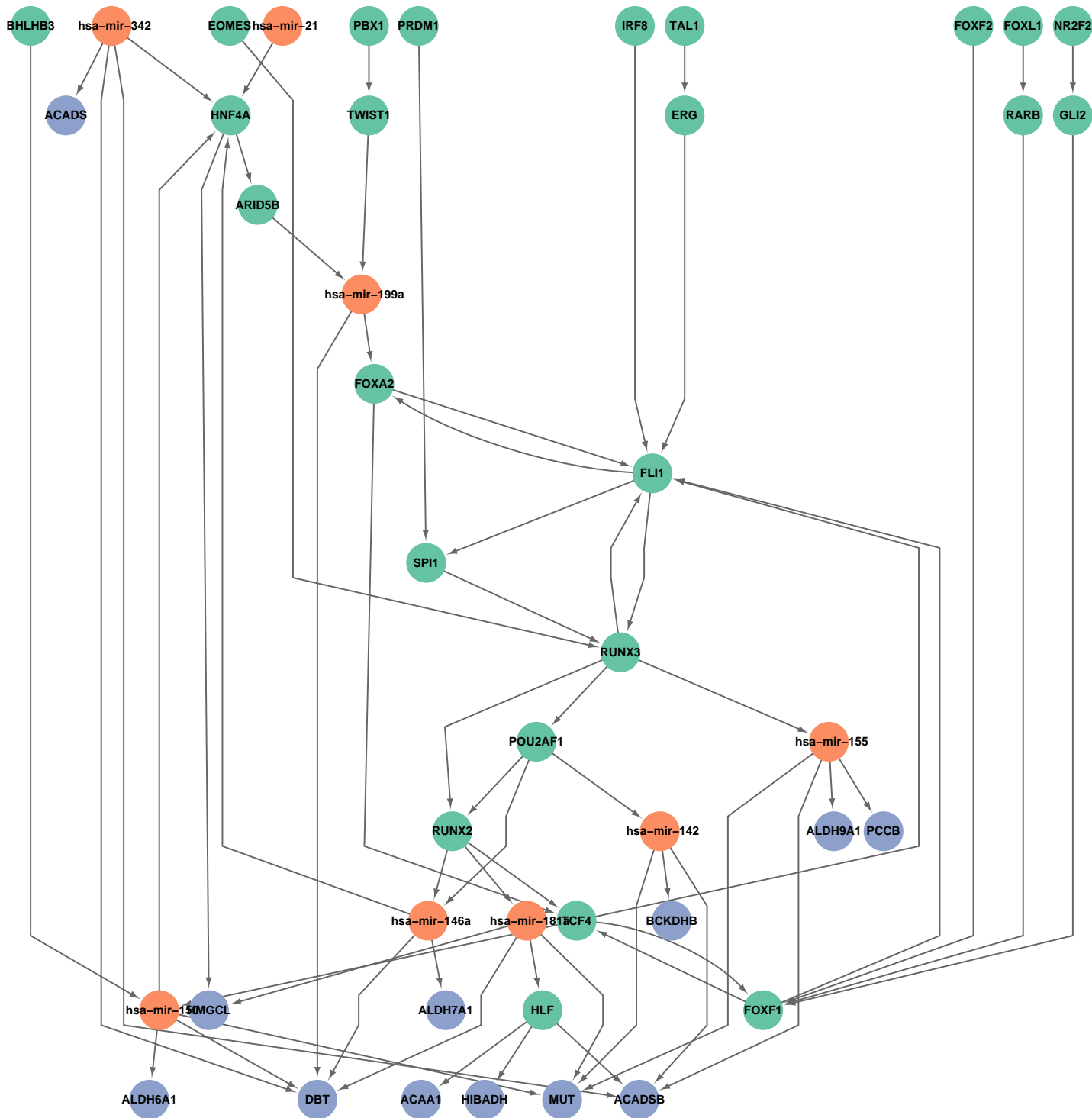

Supplement: Additional file 5: — Regulations of the enriched KEGG pathways by the core GRN. [file 1752-0509-6-32-S5.gz › regulation-on-pathway/path8.pdf]

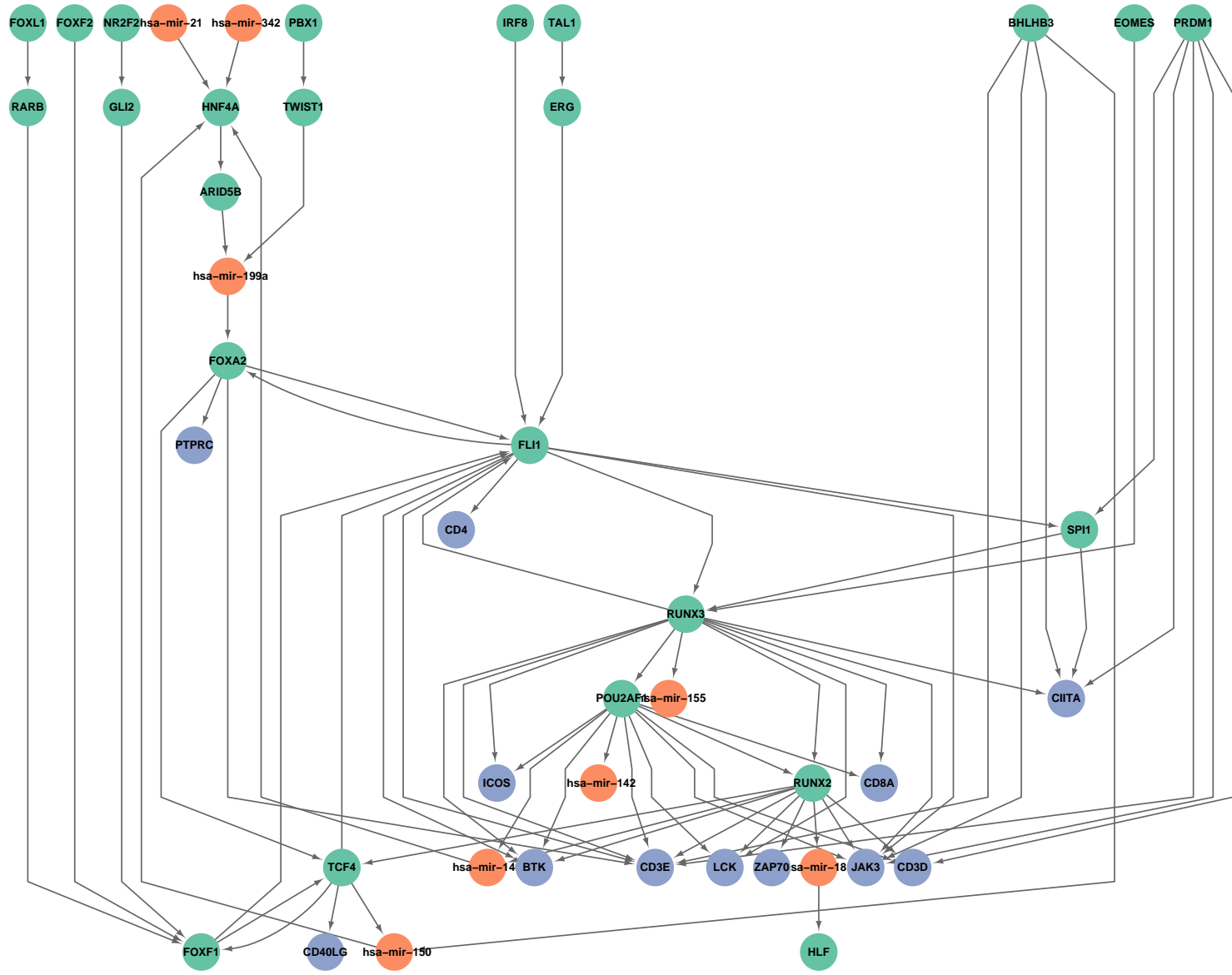

Supplement: Additional file 5: — Regulations of the enriched KEGG pathways by the core GRN. [file 1752-0509-6-32-S5.gz › regulation-on-pathway/path9.pdf]
