# Supplementary material for: Gene regulation is governed by a core network in hepatocellular carcinoma
Source: BMC Syst Biol. 2012 May 1;6:32. doi: 10.1186/1752-0509-6-32 (PMC3403900; doi:10.1186/1752-0509-6-32)
Supplement: Additional file 5: — Regulations of the enriched KEGG pathways by the core GRN. [file 1752-0509-6-32-S5.gz › regulation-on-pathway/index.html]

Regulations on the enriched KEGG pathways by core GRN


#### Regulations on the enriched KEGG pathways by core GRN

| KEGG pathway | p-value | FDR | PDF | TEXT |
| --- | --- | --- | --- | --- |
| hsa00071: Fatty acid metabolism | 1.04e-06 | 1.93e-04 | pdf | text |
| hsa04660:T cell receptor signaling pathway | 1.69e-06 | 1.56e-04 | pdf | text |
| hsa04514:Cell adhesion molecules (CAMs) | 7.74e-06 | 4.77e-04 | pdf | text |
| hsa04640:Hematopoietic cell lineage | 1.25e-04 | 5.75e-03 | pdf | text |
| hsa04512:ECM-receptor interaction | 2.42e-04 | 8.91e-03 | pdf | text |
| hsa04610:Complement and coagulation cascades | 3.18e-04 | 9.76e-03 | pdf | text |
| hsa03320:PPAR signaling pathway | 3.18e-04 | 9.76e-03 | pdf | text |
| hsa00280:Valine, leucine and isoleucine degradation | 3.90e-04 | 1.03e-02 | pdf | text |
| hsa05340:Primary immunodeficiency | 4.71e-04 | 1.08e-02 | pdf | text |
| hsa00620:Pyruvate metabolism | 4.98e-04 | 1.02e-02 | pdf | text |
| hsa04510:Focal adhesion | 6.56e-04 | 1.21e-02 | pdf | text |
| hsa00830:Retinol metabolism | 1.21e-03 | 2.02e-02 | pdf | text |
| hsa04666:Fc gamma R-mediated phagocytosis | 1.48e-03 | 2.26e-02 | pdf | text |
| hsa00640:Propanoate metabolism | 3.06e-03 | 4.27e-02 | pdf | text |

Pathway enrichment is applied by DAVID.

 Transcription factor

 microRNA

 Genes in pathways
